# The Immunogenicity of Human Senescent Cells Is Dependent on the Senescence Inducer and Cell Type

**DOI:** 10.1111/acel.70410

**Published:** 2026-02-12

**Authors:** Marie‐Lyn Goyer, Anthony Sonn, Basma Benabdallah, Oanh Le, Joshua Dulong, Joana Oschwald, Isabelle Sirois, Anca Apavaloaei, Leslie Hesnard, Jean V. Guimond, Elie Haddad, Christian Beauséjour

**Affiliations:** ^1^ Centre de Recherche du CHU Sainte‐Justine Montréal Québec Canada; ^2^ Département de Pharmacologie et Physiologie Université de Montréal Montréal Québec Canada; ^3^ Institute for Research in Immunology and Cancer (IRIC) Université de Montreal Montréal Québec Canada; ^4^ CIUSSS du Centre‐Sud‐de‐L'ile‐de‐Montréal Montréal Québec Canada; ^5^ Département de Microbiologie Immunologie et Infectiologie, Université de Montréal Montréal Québec Canada; ^6^ Département de pédiatrie, CHU Sainte‐Justine Université de Montréal Montréal Québec Canada

**Keywords:** immunogenicity, iPSC, SASP, senescence

## Abstract

Senescent cells accumulate with age and after exposure to various stresses, contributing to chronic diseases and cancer, effects largely driven by the senescence‐associated secretory phenotype (SASP). Recent evidence indicates that a subset of senescent cells exhibits immunogenic properties. However, the extent to which immunogenicity depends on the cell type and the senescence inducer remains unclear. In this study, we evaluated the immunogenic properties of various human cell types induced to senesce following exposure to ionizing radiation (IR) or oncogenic RAS. Specifically, we used human dermal fibroblasts (HDF) and induced pluripotent stem cell (iPSC)‐derived myoblasts (i‐MB), endothelial cells (i‐EC), lung progenitor cells (i‐LPC), along with immune cells from autologous donors. Our results showed that cell types exhibit a distinct SASP and express a variety of inhibitory immune ligands. Notably, senescent HDF displayed a unique immunopeptidome but failed to elicit specific immune responses from CD8+ T cells or NK cells. Similarly, senescent i‐EC and i‐LPC exhibited limited immunogenicity. In contrast, RAS‐induced senescent myoblasts demonstrated immunogenicity, characterized by T cell activation, NK cell‐mediated cytotoxicity, and immune cell recruitment in an orthotopic humanized mouse model. These findings highlight the influence of cell type and senescence inducer on immunogenicity and suggest that targeted strategies will be necessary to address the deleterious consequences of the accumulation of distinct senescent cell types.

## Graphical abstract

Using multiple cell types and autologous immune cells, the authors present the first comprehensive evaluation of the immunogenicity of human senescent cells. Their findings demonstrate that, despite exhibiting a unique immunopeptidome, senescent fibroblasts are not recognized by immune cells. Only RAS‐induced senescent myoblasts, but not RAS‐induced senescent endothelial cells or lung epithelial progenitors, were immunogenic.

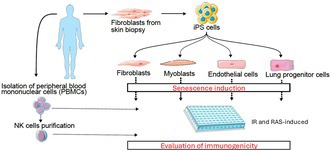

## 1 Introduction

Senescent cells accumulate in various tissues with age and in cancer survivors exposed to therapy (Marcoux et al. 2013; Suryadevara et al. 2024; Armenian et al. 2019). Their accumulation is associated with chronic age‐related diseases and cancer, primarily through their senescence‐associated secretory phenotype (SASP) (Ovadya et al. 2018; Baker et al. 2016; Childs et al. 2016). Senescent cells are known to recruit immune cells, which are believed to contribute to their elimination. However, it remains unclear why some senescent cells are immunogenic and effectively cleared, while others persist. For example, oncogene‐induced senescent hepatocytes were shown to be cleared by immune cells in mice (Kang et al. 2011; Xue et al. 2007). In contrast, senescent cells in a nevus or induced by ionizing radiation (IR) display no signs of immune clearance (Le et al. 2010; Michaloglou et al. 2005). These contrasting observations suggest that the immunogenicity of senescent cells may depend on factors such as the cell type and the senescence inducer.

It is possible that subsets of senescent cells are not immunogenic enough to be recognized and eliminated by immune cells. Alternatively, senescent cells may be sufficiently immunogenic but immune cells could become exhausted, or their cytotoxic activity impaired in the context of a systemic “aged or damaged” microenvironment (Palacio et al. 2019; Maggiorani et al. 2024; Sceneay et al. 2019). Several mechanisms have been linked to the immune evasion of senescent cells. For example, the upregulation of PD‐L1 or PD‐L2 on the surface of senescent normal and cancer cells, respectively, has been shown to limit their immunological clearance (Chaib et al. 2024; Wang et al. 2022). In mice, blocking the PD‐1/PD‐L1 interaction enhances senescent cell clearance in naturally aging mice, an effect dependent on CD8+ T cells (Wang et al. 2022). Similarly, downregulation of PD‐L1 expression in a diet‐induced obesity mouse model improved senescent cell clearance (Katsuumi et al. 2024). In addition, senescent cells can express high levels of HLA‐E or the disialylated ganglioside GD3, which, by interacting with their inhibitory receptor on NK cells and highly differentiated CD8+ T cells, protects them from immune clearance (Pereira et al. 2019; Iltis et al. 2025). Evidence indicates that senescent cells are targets of NK cells. For instance, senescent cells were shown to accumulate in perforin knockout mice (Ovadya et al. 2018). Furthermore, the immunogenicity of senescent fibroblasts diminishes over time as a result of the shedding of NKG2D‐activating ligands from their surface through the action of matrix metalloproteinases (Muñoz et al. 2019).

Despite these many immune evasive strategies, it appears that subsets of senescent cells are cleared by NK cells (Ovadya et al. 2018; Sagiv et al. 2016; Iannello et al. 2013; Ruscetti et al. 2018; Brighton et al. 2017), macrophages (Kang et al. 2011; Xue et al. 2007; Egashira et al. 2017), T cells (Hasegawa et al. 2023), or a combination of those depending on the model used. Notably, a recent study demonstrated that senescent cells overexpress MHC‐I molecules and present specific peptides that enable CD8+ T cell‐mediated immune clearance in mice (Marin et al. 2023). However, most of these immune resistance mechanisms have been identified in mice and very little is known about the immunogenicity of human senescent cells, particularly in cell types other than fibroblasts. One major reason for this lies in the difficulty to obtain various human tissues and autologous immune cells.

To overcome this limitation, we obtained human dermal fibroblasts (HDF) and peripheral blood mononuclear cells (PBMCs) from donors, generated induced pluripotent stem cells (iPSCs) from these cells, and differentiated the iPSCs into various lineages including myoblasts, endothelial cells, and lung progenitor cells. The choice of these cell lines was mostly based on our expertise in achieving successful differentiation. Using immune cells from the same donors, we then measured the immunogenicity of these senescent cell types induced either by IR or following the expression of oncogenic RAS. Our results revealed that human senescent cell types exhibit intrinsic immune‐modulatory features, including a unique immunopeptidome, expression of surface inhibitory immune ligands, and a distinct SASP. Interestingly, apart from myoblasts, the other human senescent cell types we analyzed did not display inherent immunogenicity. These findings suggest that targeted senolytics or immune‐modulatory approaches will be necessary to effectively eliminate various types of senescent cells.

## 2 Results

### 2.1 Human Senescent Fibroblasts Display a Unique Immunopeptidome

**FIGURE 1:**
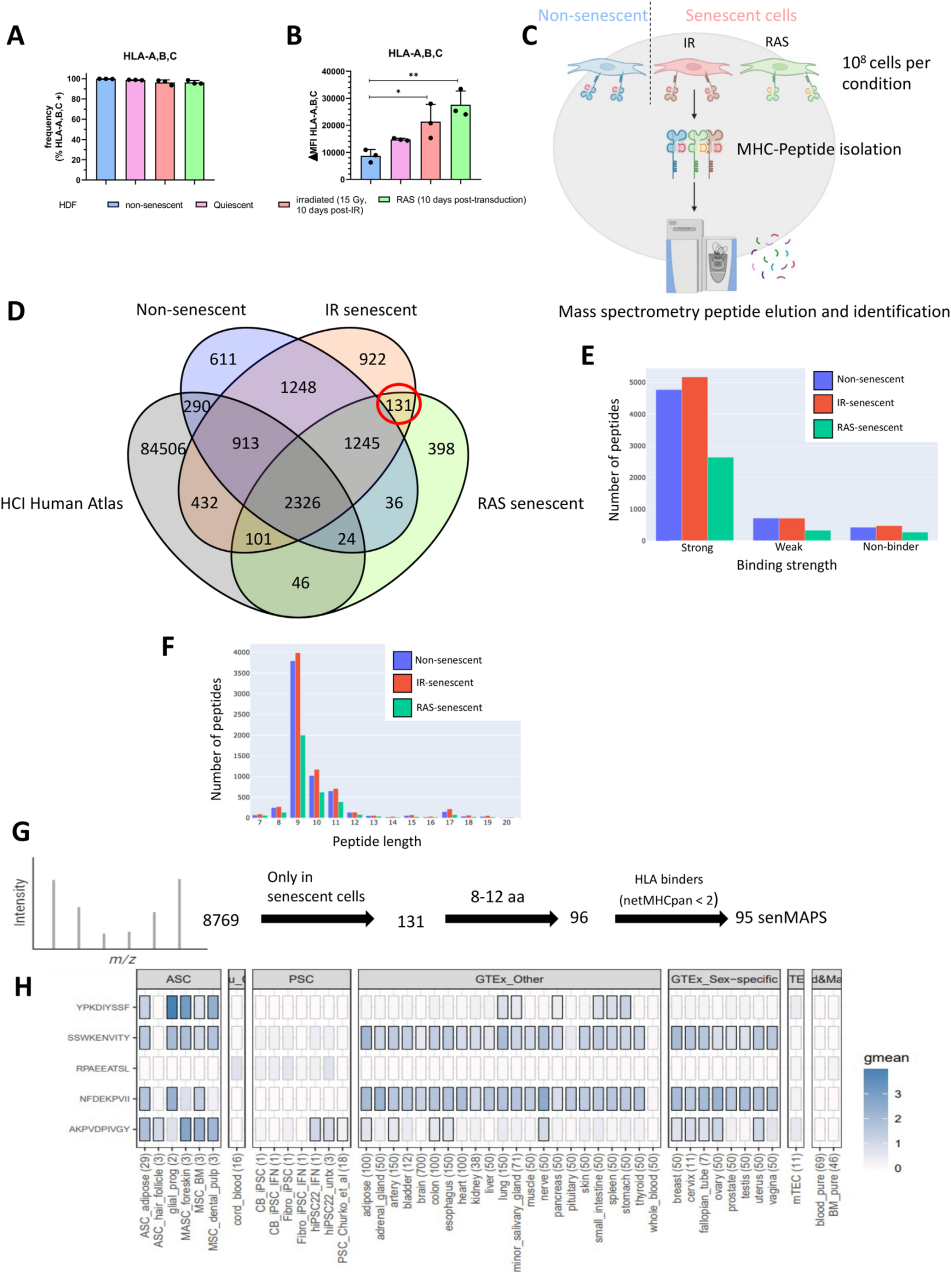
Senescent human fibroblasts display a unique immunopeptidome. (A) The proportion of non‐senescent and senescent HDF expressing HLA‐A, B, C as determined by flow cytometry. Senescence was induced by exposing HDF to ionizing radiation (15 Gy) or following RAS expression. Shown is the mean ± SD of 3 independent experiments. (B) Shown is the median fluorescence intensity (MFI) of HLA‐A, B, C on the different HDF populations. Shown is the mean ± SD of 3 independent experiments. (C) Schematic representation of the immunopeptidomics workflow on senescent and non‐senescent HDF. (D) Venn diagram showing peptides identified in non‐senescent, IR‐induced senescent, and RAS‐induced senescent HDF, along with the Human Cell Atlas (HCI). (E) Number of peptides classified as strong, weak, or non‐binders in non‐senescent, IR‐senescent, and RAS‐senescent HDF, analyzed using NetMHCpan 4.0. (F) Numbers of peptide identified based on size. (G) Identification of potential senMAPs based on the indicated exclusion criteria. (H) Expression of five specific senMAPs was analyzed across RNA‐seq datasets of 47 different human tissues, with selection criteria including their lack of expression in medullary thymic epithelial cells (mTEC).

To assess the immunogenicity of senescent HDF, we first measured the expression of MHC‐I proteins at the surface of non‐senescent and senescent HDF using flow cytometry. Senescence was induced by exposing HDF to ionizing radiation (IR) at the dose of 15 Gy or by ectopic expression of oncogenic RAS, two well‐characterized inducers we previously used on these cells (Goyer et al. 2024). In opposition to MHC‐II which is not expressed by HDF, MHC‐I was found on nearly all cells and its expression increased in senescent HDF (Figure 1A,B). Given the role of MHC‐I in presenting peptides to CD8 T cells, we then established the immunopeptidome profile of non‐senescent and senescent HDF using mass spectrometry (Figure 1C). Analysis of peptides eluted from immunoprecipitated MHC‐I revealed 8769 unique peptides of which only 131 were detected in senescent HDF but not in non‐senescent HDF or in the “HLA Ligand Atlas” (Figure 1D and Table S4). The latter consists of more than 90,428 MHC‐I (51 alleles) from 227 ligandomes samples spanning 29 different benign tissues obtained from 21 human subjects (Marcu et al. 2021). Each peptide was further analyzed based on size and predicted binding affinity to MHC‐I proteins. Only peptides in the range of 8 to 12 amino acids (the length compatible with MHC‐1 binding) and those predicted to be strong binders using netMHCpan were considered for further analysis (Figure 1E,F). These steps narrowed down our list of senescence‐associated MHC class I peptides (or senMAPs) to 95 (Figure 1G).

We then applied a bioinformatic algorithm to identify peptides whose coding sequences show low expression (below 8.55 reads per hundred million) in various healthy tissues, including in thymic epithelial cells (mTECs), and which have a higher likelihood of generating a specific adaptive immune response (Apavaloaei et al. 2022). The expression of our 95 senMAPs was compared to data from the Genotype‐Tissue Expression (GTEx) database, which includes 29 healthy tissues, as well as RNA‐seq data from 43 adults and 36 pluripotent stem cell samples, 69 whole blood, 46 bone marrow, 16 umbilical cord blood samples, and 11 mTEC samples. Interestingly, 5 of our peptides (YPKDIYSSF, SSWKENVITY, RPAEEATSL, NFDEKPVII, and AKPVDPIVGY) had a frequency of less than 1 read per hundred million in mTECs (Figure 1H and Table S1). The RPAEEATSL peptide was discarded as it codes for the telomerase enzyme which was used to immortalize HDF. These results suggest that senescent HDF possess a unique immunopeptidome not presented during thymic T cell selection, potentially capable of triggering a specific immune response.

### 2.2 No Evidence That Senescent Human Fibroblasts Are Immunogenic

**FIGURE 2:**
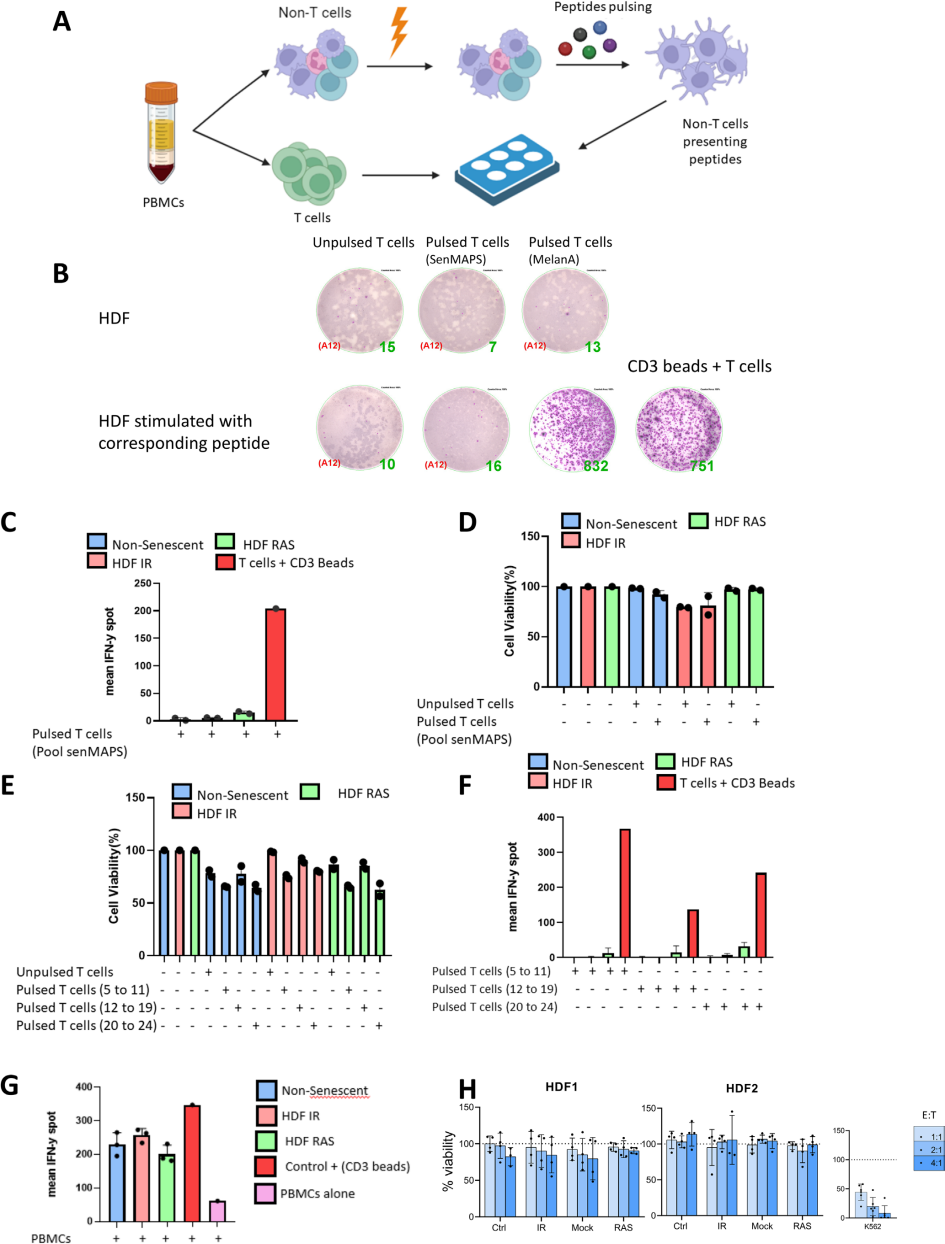
Senescent human fibroblasts are not the target of immune cells. (A) Schematic representation of the FEST assay. PBMCs autologous to HDF were separated into T cells and non‐T cells. The non‐T fraction was irradiated (30 Gy), pulsed with peptides, and presented to T cells for 20 days. (B) ELISpot assay detecting in control HDF or those stimulated with peptides and challenged by peptide‐specific T cells (5:1 T cell:HDF ratio). CD3+ beads (10 μg/mL) served as a positive control. Shown are spot counts from one experiment that is representative of three independent biological replicates. (C) Quantification of IFN‐γ production for non‐senescent, IR‐senescent, or RAS‐senescent HDF challenged with senMAPS‐pulsed T cells. Shown are results of one experiment (mean ± SD of *n* = 2) representative of three independent biological replicates. (D) HDF cell viability quantified by flow cytometry following coculture with unpulsed or senMAPS‐pulsed T cells (5:1 T cell:HDF ratio). Viability was determined by comparing cocultured cells to controls (target cells only). Shown are results of one experiment (mean ± SD of *n* = 2) representative of three independent biological replicates. (E) HDF viability assessed after coculture with T cells pulsed with the indicated peptides pools. Flow cytometry was used to quantify viable target cells compared to control wells. Shown are results of one experiment (mean ± SD of *n* = 2) representative of three independent biological replicates. (F) Quantification of IFN‐γ production from non‐senescent, IR‐induced senescent, or RAS‐induced senescent HDF challenged by pulsed T cells stimulated with the indicated peptide pools. Shown are results of one experiment (mean ± SD of *n* = 2) representative of three independent biological replicates. (G) Quantification of IFN‐γ production from non‐senescent, IR‐induced senescent, or RAS‐induced senescent HDF challenged with PBMCs (5:1 PBMC:HDF ratio) or CD3+ beads. Shown are results of one experiment (mean ± SD of *n* = 3) representative of three independent biological replicates. (H) Cell viability of HDF or K562 (positive control) assessed after challenge with NK cells at varying effector‐to‐target (E:T) ratios. Two HDF donor cell lines were tested, with results shown as mean ± SD from four independent biological replicates for each HDF donor.

To verify the immunogenicity of our senMAPs, we used PBMCs collected from the same donor from whom the HDF were isolated for mass spectrometry analysis. Given the expected low frequency of specific T cells in blood capable of recognizing senMAPs in the absence of prior immunization, we opted to perform a Functional Expansion of Specific T cells (FEST) assay. In this assay, T cells are purified from freshly isolated PBMCs, and the remaining immune cells are irradiated to serve as antigen‐presenting cells. Non‐irradiated purified T cells are then incubated twice for 10 days (for a total of 20 consecutive days) with the antigen‐presenting cells, either pulsed with a pool of senMAP peptides or with a MelanA peptide (used as a positive control) or left unpulsed. This procedure is designed to trigger the expansion of rare T cell clones capable of recognizing specific peptides (Figure 2A).

Expanded T cells were then co‐cultured at a 4:1 ratio for 24 h with autologous non‐senescent HDF which had been previously pulsed or left unpulsed with the corresponding peptides. Using an IFN‐y ELISpot assay, we did not observe activation of T cells against our senMAPs (Figure 2B). In contrast, MelanA‐pulsed T cells were fully activated when co‐cultured with MelanA peptide‐pulsed HDF (Figure 2B). To rule out the possibility of inefficient peptide presentation by HDF, we repeated the ELISpot assay using expanded T cells co‐cultured with either non‐senescent or senescent HDF; however, no T cell activation was observed (Figure 2C). Additionally, pulsed expanded T cells were ineffective at killing senescent HDF in a direct cytotoxicity assay (Figure 2D).

We then tested an additional set of 20 senMAPs that also showed low expression in mTECs (see Table S1). Once again, these peptides, organized into distinct pools, failed to activate autologous expanded T cells, as determined by ELISpot assays and direct cytotoxicity against HDF (Figure 2E,F). As expected, we also did not detect specific activation of immune cells, as measured by CD69 expression in T cells and ELISpot assays, when incubating senescent HDF with autologous whole PBMCs (Figure 2G).

We next tested the immunogenicity of senescent HDF against peripheral blood‐derived activated NK cells. We found that NK cells did not preferentially kill senescent HDF at any of the effector‐to‐target ratios tested and that senescent HDF were even protected from cell lysis depending on the experimental conditions (Figure 1H and Figure S1). In contrast, NK cells were fully capable of killing K562 cancer cells, which are used as a positive control for cell lysis given they lack the expression of MHC‐I, a strong inhibitory ligand of NK cells' activation (Figure 1H). The expression of several negative regulators of immune cells' activation was increased at the surface of senescent HDF (Figure S2), and it is possible that one or more of these regulators played a role in protecting senescent HDF from NK cell‐mediated killing. Overall, these results suggest that neither the most promising senMAPs nor senescent HDF themselves are effectively targeted by NK or T cells in vitro.

### 2.3 Human Senescent Endothelial Cells Are Not the Target of Immune Cells In Vitro

We next sought to determine whether human cell types other than fibroblasts would become immunogenic following the induction of senescence. To work in an autologous setting, we chose to use iPSC‐derived endothelial cells (hereafter referred to as i‐EC), as we had access to peripheral blood from the same donors used to generate the iPSC lines. First, we differentiated iPSCs into i‐EC and confirmed their phenotype by the expression of von Willebrand factor (vWF) and their ability to form capillary‐like structures following enrichment of the CD31+/CD144+ population (Figure S3A–C). Senescence induction in i‐ECs from two donors was validated by demonstrating proliferation arrest and by senescence‐associated beta‐galactosidase (SA‐β‐gal) staining after either overexpression of oncogenic RAS or exposure to 8 Gy irradiation (Figure S3D–F).

**FIGURE 3:**
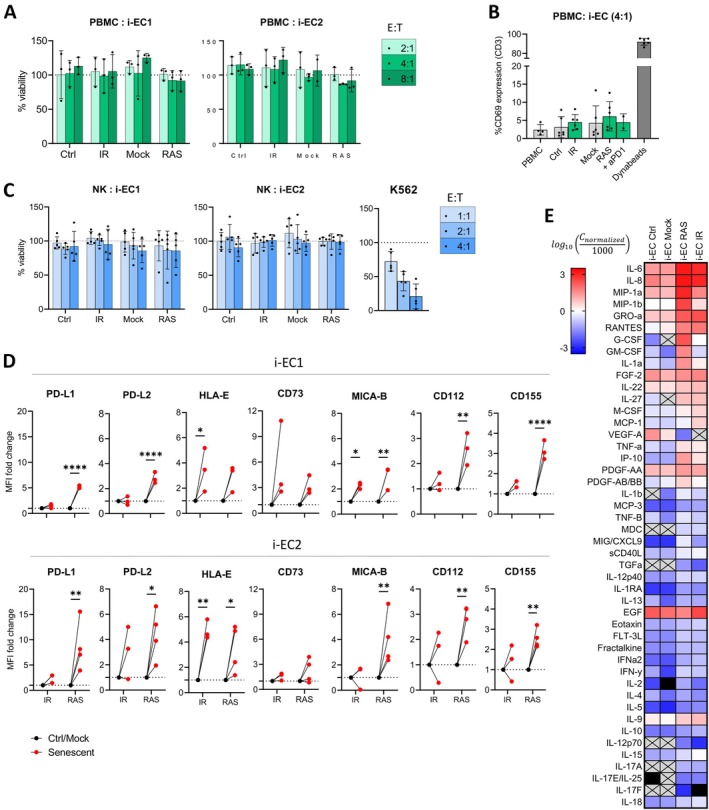
Human senescent endothelial cells are not the target of immune cells in vitro. (A) Viability of control (non‐senescent or mock transduced) or senescent (RAS or IR‐induced) i‐EC populations from two donors after 24 h of coculture with autologous PBMCs at varying effector (E) to target (T) cell ratios. Percentage of cell viability was assessed by comparing the amount of viable target cells in coculture compared to control wells containing only target cells (no effectors). Cell counts were determined using flow cytometry. Shown is the mean ± SD of *n* = 3 independent biological experiments. (B) Proportion of CD3+ cell subset expressing CD69 as determined by flow cytometry after 24 h of coculture at a ratio of 4 PBMC to 1 i‐EC. Mean ± SD of *n* = 6 independent biological experiments. Data from both donors were pooled. (C) Viability of i‐EC from two donors after 24 h of coculture with NK cells at varying effector (E) to target (T) cell ratios. K562 serves as a positive control of cytotoxicity. Shown is the mean ± SD of *n* = 5 independent biological experiments. Cell counts were determined using flow cytometry. (D) Mean fluorescence intensity (MFI) fold change as determined by flow cytometry of various immune surface ligands from senescent i‐EC or their respective controls. Shown is the mean of *n* = 3 independent biological experiments. Satistical analysis was performed using two‐way ANOVA followed by Šidák's multiple comparisons test. (E) Heatmap of cytokines detected from a multiplex assay performed using conditioned media collected from i‐EC 10 days after senescence induction. Gray squares marked with an X indicate an undetected value. Each square represents the average value of *n* = 4 different samples, with 2 samples taken from each donor. *p < 0.05, **p < 0.01, ****p < 0.0001.

When co‐cultured with autologous PBMCs, senescent i‐EC were not killed and failed to activate T cells, as indicated by the lack of CD69 expression on the CD3+ T cell population (Figure 3A,B). Similarly, senescent i‐EC were not killed by NK cells in vitro (Figure 3C). Senescent i‐EC upregulated the DNAM ligands CD112 and CD155 as well as the inhibitory ligand HLA‐E (Figure 3D). However, unlike senescent HDF, they upregulated the activating ligands MICA/MICB (Figure 3D). As expected, senescent i‐EC, regardless of the inducer, secreted a pro‐inflammatory SASP, although it was distinct from that observed in HDF (Figure 3E and Figure S4). In this context, we hypothesize that, as we previously observed with HDF, the SASP and the expression of inhibitory ligands create an overall hostile environment that protects senescent i‐EC from immune cell‐mediated clearance (Palacio et al. 2019; Cruz‐Barrera et al. 2025).

### 2.4 Human Senescent Lung Progenitor Cells Are Not Immunogenic

**FIGURE 4:**
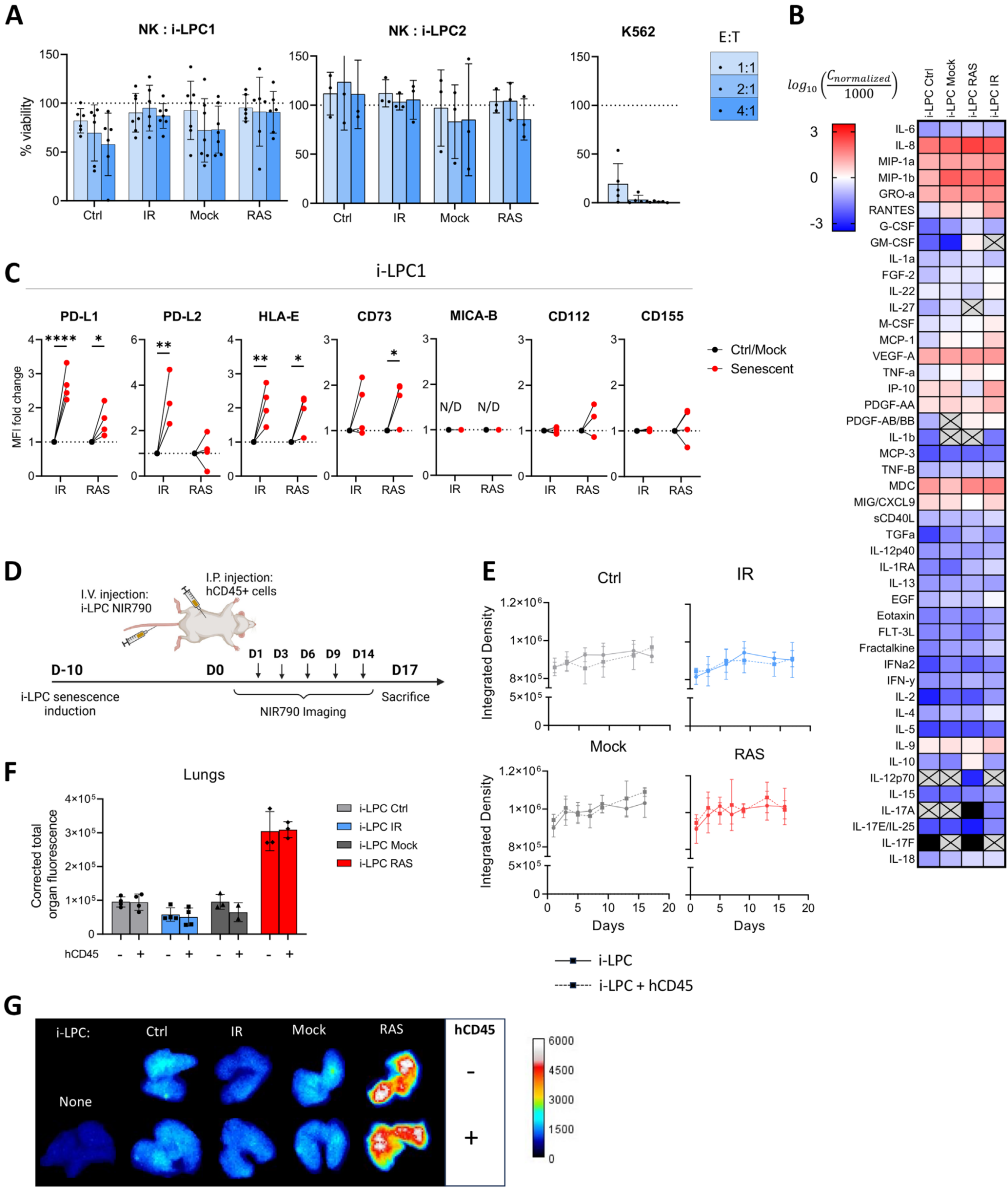
Human senescent lung progenitor cells are not immunogenic. (A) Viability of control (non‐senescent or mock transduced) or senescent (RAS or IR‐induced) i‐LPC populations from two donors after 24 h of coculture with NK cells at varying effector (E) to target (T) cell ratios. K562 serves as a positive control of cytotoxicity. Shown is the mean ± SD of *n* = 6 (i‐LPC1) or *n* = 3 (i‐LPC2) independent biological experiments. Cell counts were determined using flow cytometry. (B) Heatmap of cytokines detected from a multiplex assay performed using conditioned media collected from i‐LPC 10 days after senescence induction. Gray squares marked with an X indicate an undetected value. Each square represents the average value of *n* = 4 different samples, with 2 samples taken from each donor. (C) Mean fluorescence intensity (MFI) fold change as determined by flow cytometry of various immune surface ligands from senescent i‐LPC or their respective controls. Shown is the mean of *n* = 4 independent biological experiments. Data analysis was performed using two‐way ANOVA followed by Šidák's multiple comparisons test. (D) Schematic representation of the intra‐veinous (I.V.) injection of control or senescent i‐LPC labeled with NIR790 and the intra‐peritoneally (I.P.) injection of human autologous CD45+ immune cells (PBMCs and granulocytes 5 × 10^6^ each) on Day 0. Persistence of fluorescent cells was measured every 3 days (indicated by arrows) until the animals were sacrificed on Day 17. (E) Average integrated density values of the NIR790 signal measured over time from mice having received i‐LPC with or without immune cells. (F) Average integrated density values of the NIR790 signal from the lungs imaged ex vivo at sacrifice. The signal was corrected for organ area and normalized to the baseline signal from lungs that did not receive i‐LPC. *n* = 4 lungs collected from an individual Ctrl or IR mouse, *n* = 3 for Mock and RAS. (G) Representative images showing the NIR790 signal from the lungs at sacrifice. N/D, not detected, *p < 0.05, **p < 0.01, ****p < 0.0001.

We next aimed to evaluate whether senescent lung progenitor cells would respond differently to immune cells. We first differentiated our iPSC lines into lung progenitor cells (i‐LPC) and then confirmed that i‐LPC, when cultured in 3D Matrigel, could form spheres composed of cells expressing NKX2‐1 and surfactant protein C, both specific markers for alveolar type II cells (AT2) (Figure S5A,B). However, these cells were not fully differentiated into AT2 cells, as differentiation was intentionally halted at the progenitor stage to allow for their continued proliferation. Senescent i‐LPC exhibited increased SA‐β‐gal activity and ceased proliferation upon transduction with RAS or exposure to 7 Gy IR (Figure S5C–E). A lower dose of IR was necessary to prevent i‐LPC from undergoing apoptosis. When co‐cultured with activated NK cells for 24 h, senescent i‐LPC were not preferentially killed compared to non‐senescent control cells (Figure 4A).

Notably, the secretion of SASP‐related molecules from senescent i‐LPC was not as elevated as in other senescent cell types, except for IL‐10 and IL‐4 (in RAS‐induced and IR‐induced senescent cells respectively, two cytokines known to negatively impact immune cells (Figure 4B; Figure S4)). As observed with HDF and i‐EC, senescent i‐LPC also displayed strong expression of cell surface inhibitory markers such as HLA‐E and PD‐L1 (Figure 4C).

To determine whether senescent i‐LPC exhibit immunogenicity towards autologous PBMCs, we used an in vivo humanized mouse model to avoid the presence of Matrigel which is necessary for sphere formation in vitro. We stained i‐LPC with the NIR790 dye and injected the cells into the tail vein of NSG‐SGM3 mice leading to the engraftment of i‐LPC primarily in the lungs (Figure 4D). On the same day, 10 million autologous human white blood cells (PBMCs and granulocytes—5 million each) were injected intraperitoneally. Live imaging of the NIR790 signal, up to 17 days post injection revealed weak but equivalent signal in all groups of mice, suggesting senescent cells are not preferentially eliminated by immune cells (Figure 4E). NIR790 signal detected in the lungs at the time of sacrifice showed no significant differences between immune‐deficient and following the adoptive transfer of immune cells (Figure 4F,G). Intriguingly, RAS‐induced senescent i‐LPC consistently showed much higher NIR790 signal, despite the cells being confirmed not to proliferate prior to their injection (Figure S5). We believe these cells are more resistant to cell death by anoikis as the result of RAS expression (Mason et al. 2016). Noteworthy, we previously demonstrated that the adoptive transfer of 10 million immune cells is sufficient to induce immune rejection of non‐differentiated iPSCs and allogenic differentiated cells (Benabdallah et al. 2019, 2021). Therefore, it is unlikely that the persistence of senescent i‐LPC is due to an insufficient number of injected human immune cells. These results indicate that human senescent i‐LPC are not eliminated by immune cells.

### 2.5 RAS‐Induced Senescent Human Myoblasts Are Immunogenic

**FIGURE 5:**
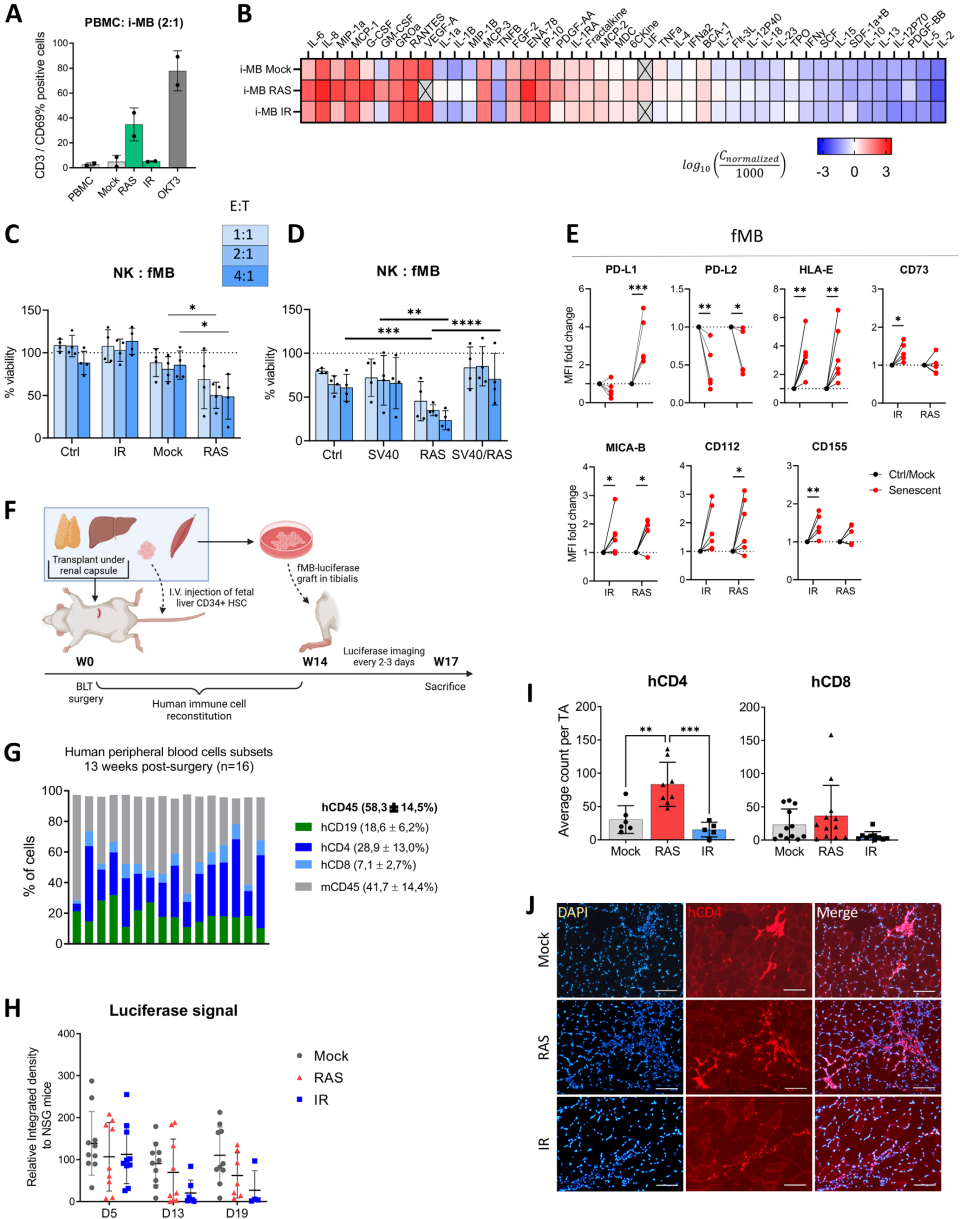
RAS‐induced senescent human myoblasts are immunogenic. (A) Proportion of CD3+ cell subset expressing CD69 as determined by flow cytometry after 24 h of coculture with autologous PBMCs at a ratio of 2 PBMCs to 1 i‐MB. Mean ± SD from *n* = 2 experiments are shown. (B) Heatmap of cytokines detected from a multiplex assay performed using conditioned media collected from i‐MB 10 days after senescence induction. Gray squares marked with an X indicate an undetected value. Each square represents the average value of *n* = 3 different samples. (C, D) Viability of the indicated fMB populations after 24 h of coculture with NK cells at varying effector (E) to target (T) cell ratios. K562 serves as a positive control of cytotoxicity. Shown is the mean ± SD of *n* = 4 experiments performed in triplicate. Cell counts were determined using flow cytometry. Satistical analysis was performed using two‐way ANOVA followed by Fisher's LSD test. (E) Mean fluorescence intensity (MFI) fold change as determined by flow cytometry of various immune surface ligands from senescent fMB compared to their respective controls. Shown is the mean of *n* = 5–6 experiments performed in triplicate. Satistical analysis was performed using two‐way ANOVA followed by Šidák's multiple comparisons test. (F) Schematic timeline for the establishment of the BLT mouse model following the injection of autologous human fetal tissues. On Week 14, autologous fMB (expressing the luciferase transgene) were injected in the tibialis and luminescence detected every 2–3 days until the animals were sacrificed 21 days later (week 17). (G) Proportion of different human immune cells subsets and mouse CD45+ cells determined by flow cytometry in blood samples collected from BLT mice on week 13. *n* = 16 mice. (H) Average integrated density values of the luciferase signal at Day 5, 13 and 19 from mouse tibialis grafted with mock or senescent fMB. Integrated density value is normalized to the average signal from NSG mice (which have no human immune cells) for each group. (I) Average count of hCD4 and hCD8 cells determined from immunofluorescence staining from five slices areas showing the injection site. *n* = 5–12 TA muscles. Satistical analysis was performed using a one‐way ANOVA followed by Šidák's multiple comparisons test. (J) Representative immunofluorescence images of hCD4 infiltration in BLT mice tibialis injected with fMB from each group. BLT, Bone marrow, liver, thymus; fMB, Fetal myoblasts; i‐MB, IPSC‐derived myoblasts; TA, Tibialis anterior. *p < 0.05, **p < 0.01, ***p < 0.001. ****p < 0.0001.

Finally, given the increasing evidence showing the impact of senescence on myogenesis, we evaluated the immunogenicity of either iPSC‐derived myoblasts (i‐MB) or primary myoblasts obtained from fetal tissue (fMB). We differentiated human iPSC into i‐MB using a defined myogenic medium and overexpression of the transcription factor MyoD, which induces a terminally differentiated and quiescent cellular state (Figure S6A) (Benabdallah et al. 2021). When compared to freshly dissociated fMB, i‐MB expressed equivalent myogenic markers, such as CD56, Desmin, and Myosin heavy chain (MyoHC) (Figure S6B,C). Exposure of fMB or i‐MB to RAS viral particles or 12 Gy IR led to their senescence, as determined by increased SA‐β‐gal activity and halted proliferation (Figure S6D–F). Surprisingly, co‐culture of RAS‐induced, but not IR‐induced, senescent i‐MB with autologous PBMCs resulted in enhanced T cell activation, as determined by CD69 expression (Figure 5A). We also observed a more pronounced pro‐inflammatory SASP in RAS‐induced compared to IR‐induced senescent i‐MB (Figure 5B).

We next aimed to confirm the enhanced immunogenicity of i‐MB using primary fetal myoblasts, which have the advantage of proliferating in vitro (in comparison to i‐MB that are quiescent), allowing for their expansion and further characterization. We first measured the immunogenicity of fMB using NK cells collected from healthy donors. We observed that RAS‐induced, but not IR‐induced, senescent fMB were lysed by activated NK cells at 2:1 and 4:1 effector‐to‐target ratios (Figure 5C). To determine if the observed immunogenicity was due to the senescence phenotype or specific to RAS expression, we produced fMB resistant to RAS‐induced senescence by also expressing the Simian Virus 40 large T antigen (SV40). The large T antigen prevents senescence induction by binding and inactivating the p53 and retinoblastoma proteins (Beausejour et al. 2003). We found that fMB expressing SV40 alone or in combination with RAS were not immunogenic (Figure 5D). As fMB expressing both SV40 and RAS are actively proliferating, these results suggest that the immunogenicity observed with RAS alone is due to the specific senescence phenotype of fMB. Such immunogenicity was surprising given that, like all other cell types tested so far, senescent fMB also exhibited a marked increase in the expression of several immunoregulatory receptors, such as HLA‐E and PD‐L1, with the exception of PD‐L2, whose expression was decreased (Figure 5E).

Furthermore, as PBMCs autologous to fetal tissues were not available, we employed the BLT mouse model, where a human immune system is reconstituted in mice following the engraftment of fetal tissues autologous to the previously mentioned fMB (Figure 5F,G). Notably, BLT mice do not develop NK cells but have functional T cells due to the presence of the implanted human thymus tissue (Chuprin et al. 2023). We injected luciferase‐expressing fMB into the tibialis anterior (TA) muscle of BLT and control NSG mice. We observed no significant cell clearance of senescent fMB over time in all groups of mice (Figure 5H). However, there was a notable increase in CD4 T cell infiltration in TA muscles grafted with RAS‐induced senescent fMB (Figure 5I,J). Together, these findings suggest that RAS‐induced senescent myoblasts exhibit heightened immunogenicity, characterized by T cell activation in vitro and recruitment in vivo, as well as NK cell‐mediated cytotoxicity.

## 3 Discussion

In this study, we used autologous expanded T cells, NK cells, and whole PBMCs to show that senescent human cells, whether dissociated from tissues or differentiated from iPSCs, display a different immunogenicity profile based on the cell type and senescence inducer. We found that senescent HDF are not immunogenic, despite having a unique immunopeptidome. Indeed, we identified 95 unique senMAPs common to both IR‐induced and RAS‐induced senescent HDF. However, none of the 24 senMAPs tested were able to activate expanded T cells in vitro, despite increased MHC‐I expression on senescent HDF. We cannot exclude the possibility that the frequency of circulating T cells capable of recognizing senMAPs was too low in the absence of prior immunization, as previously demonstrated using mouse fibroblasts (Marin et al. 2023). Additionally, it is well known that the immunopeptidome is largely dependent on the cellular context (Caron et al. 2011), so it is possible that immunogenic senMAPs could exist and be identified if we had used another donor or cell type. The large number of cells required for immunopeptidomic studies is, however, a limitation when using primary human cells.

Moreover, the fact that senescent HDF were not preferentially killed by NK cells was surprising, given that others have shown otherwise (Muñoz et al. 2019; Sagiv et al. 2016). Such disparity may be explained by the fact that our senescent HDF did not show a significant upregulation of the NKG2D ligands MICA/B, in contrast to the upregulation of inhibitory ligands such as PD‐L1, PD‐L2, or HLA‐E, as previously reported by others (Chaib et al. 2024; Wang et al. 2022; Pereira et al. 2019; Majewska et al. 2024). The source of NK cells is unlikely to be a defining factor, as we never observed increased killing of senescent HDF regardless of the donor (data not shown). Noteworthy, only IR‐induced senescent HDF derived from iPSCs (hereafter referred to as i‐HDF) were preferentially killed by NK cells (Figure S7). This enhanced immunogenicity of i‐HDF may be explained by our previous finding that these cells have increased sensitivity to stress and secrete a more potent pro‐inflammatory SASP compared to their parental senescent autologous counterpart (Goyer et al. 2024). Notably, we did not observe increased sensitivity to NK cells using either IR‐induced senescent i‐EC or i‐LPC cells, suggesting this may be an exclusive feature of i‐HDF.

Strikingly, when we evaluated the cytotoxicity of NK cells against HDF at a higher cell density (20,000 vs. 5000 cells per well) while maintaining the same effector‐to‐target ratio, we observed that senescent HDF were protected from NK cell lysis. Indeed, we observed that long‐term (7 days) IR‐induced senescent HDF were protected against NK cell‐mediated cytotoxicity, in contrast to short‐term (3 days) IR‐induced senescent HDF (Figure S1). We believe the size difference between non‐senescent and senescent HDF is no longer a factor when cells are seeded at a high density, which facilitates the interaction between NK cells and their targets. These results are consistent with previous reports showing that senescent fibroblasts can protect themselves by either upregulating HLA‐E, the ganglioside GD3 or by cleaving activating ligands (Pereira et al. 2019; Iltis et al. 2025; Muñoz et al. 2019). It is unlikely the expression of PD‐L1 had a role in protecting senescent HDF as the NK cells we used did not express PD‐1. Moreover, blocking PD‐1 with nivolumab did not increase the cytotoxicity of expanded T cells against senescent HDF (data not shown).

Endothelial cells and lung epithelial cells have been shown to undergo senescence, contributing to vascular aging, atherosclerosis and lung diseases (Childs et al. 2016; Roos et al. 2016; Yamada et al. 2022; Tian et al. 2019). In mice, previous studies have demonstrated that NK cells play a critical role in the perforin‐mediated clearance of naturally occurring senescent cells in lungs and other tissues (Ovadya et al. 2018). However, it remains largely unknown whether human lung progenitor and endothelial senescent cells are cleared by immune cells. Our results with senescent i‐EC and i‐LPC demonstrate that despite having a distinct SASP, these cells are not preferentially cleared by NK cells or autologous immune cells. As observed with senescent HDF, the lack of immunogenicity in i‐EC and i‐LEC may also be related to their strong expression of negative immunoregulatory molecules.

The impact of senescence on the muscle tissue is only beginning to emerge. The accumulation of senescent myoblasts was recently confirmed in samples collected from myotonic dystrophy type 1 patients (Conte et al. 2023). Elimination of these cells using a senolytic drug rescued their proliferation and differential potential. However, immune surveillance of damaged muscle cells is largely undefined. Of the four cell types tested in this study, only RAS‐induced senescent myoblasts were killed by NK cells and could activate autologous T cells in vitro. Nonetheless, aberrant oncogenic signaling is a rare physiological occurrence in muscle, and thus the immunological clearance of oncogene‐induced senescent cells and its potential physiological impact in muscle remain to be determined.

Independently of the cell type, we observed that RAS‐induced senescence generated a stronger and distinct SASP compared to IR‐induced senescence, with the overall SASP profile being unique to each cell type analyzed. To what extent, in a humanized setting, the SASP contributes to immune cell recruitment and eventual senescent cell clearance remains to be determined. On the other hand, the SASP may dampen the immune response, as we previously observed in mice (Palacio et al. 2019). We believe the impact of the SASP is highly dependent on the environment, both in the context of chronological aging and in cancer therapy‐induced senescence (Faget et al. 2019).

Ultimately, our findings suggest that the immunogenicity of human senescent cells is partially influenced by intrinsic factors related to their tissue of origin and the mechanism they deploy for protection. Further studies are needed to pinpoint the specific contributions of these mechanisms and provide insights for therapeutic immune strategies targeting senescent cells.

## 4 Material and Methods

### 4.1 Cell Culture, Reprogramming, and Differentiation

HDF and PBMCs were obtained from skin biopsies and blood samples collected from healthy donors after informed consent was obtained in accordance with the ethics committee from the Centre Hospitalier Universitaire Sainte‐Justine (protocol 2017–1476). HDF or PBMCs were used by the iPSC reprogramming core facility at CHU Sainte‐Justine for iPSC generation and subsequent differentiation. Protocols for cell reprogramming, differentiation into fibroblasts (i‐HDF), and cell characterization have been previously described (Goyer et al. 2024). HDF and i‐HDF were cultured in DMEM (Multicell) supplemented with 10% fetal bovine serum (FBS) (Gibco) and 1% penicillin/streptomycin (Multicell). For mass spectrometry, where a large number of HDF were required, HDF from donor 1 were immortalized using lentiviral particles carrying the catalytic subunit of the telomerase enzyme (HDF‐hTERT) as previously described (Beausejour et al. 2003).

iPSC‐derived myoblasts (i‐MB) were differentiated by first culturing iPSC colonies in MB1 myogenic medium (Hyclone) supplemented with 10 ng/mL of basic fibroblast growth factor (bFGF) (Peprotech) for five days on Geltrex‐coated (Gibco, A14132‐02) culture dishes. Cells were maintained in culture with MB1 medium before being transduced with a MyoD‐expressing adenovirus for 5 h at an MOI of 30 as previously described (Benabdallah et al. 2021). This transduction induced a terminally differentiated, non‐proliferative state. Fetal myoblasts (fMB) were obtained from fetal muscle tissues of a healthy donor after surgical abortion at approximately week 20 of pregnancy (approved by the ethical committee of CHU Sainte‐Justine, CER#2126). Tissues were minced into small pieces and digested using a solution of phosphate‐buffered saline (PBS) containing 0.2% collagenase (Roche) and 0.25% dispase (STEMCELL Technologies) at 37°C for 30 min, with manual intermittent mixing. Cells were cultured in MB1 medium before in vivo assays or Sk MAX–supplemented medium (Multicell, 301–060) containing 20% FBS and Primocin (InvivoGen, ant‐pm‐05). Cells were passaged every 4 days using 0.05% trypsin (Gibco).

iPSC‐derived endothelial cells (i‐EC) were obtained by first inducing mesoderm differentiation in iPSC colonies cultured in APEL2 medium (STEMCELL Technologies) supplemented with 6 μM CHIR99021 (STEMCELL Technologies) and 25 ng/mL BMP4 (Peprotech) for the first 2 days. The medium was then supplemented with 50 ng/mL VEGF (Gibco), 25 ng/mL BMP4, and 20 ng/mL bFGF for 4 days. CD31+/CD144+ cells were then selected by FACS and expanded in EGM2‐supplemented medium (Lonza, CC‐3162). Cells were passaged every 4 days using TrypLE (Gibco) and plated on Geltrex‐coated culture dishes.

Differentiation of iPSC into lung progenitor cells (i‐LPC) followed the protocol described by Jacob et al. (Jacob et al. 2019) using serum‐free medium described in Table S2. Cells were maintained in culture after sorting for NKX2‐1+/CD47hi/CD26lo populations. They were grown in 3D Matrigel droplets to form alveolospheres, with complete serum‐free differentiation medium replenished every 2–3 days. Cells were passaged weekly by dissolving the 3D Matrigel with 2 mg/mL dispase, followed by dissociation of the alveolospheres into single cells using 0.05% trypsin for 15 min. To prevent cell death, 10 μM ROCK inhibitor (STEMCELL Technologies) was added to the medium for the first 48 h after each passage.

### 4.2 Senescence/Quiescence Induction and Detection

Quiescence was induced by culturing cells to confluency or by serum starvation in DMEM containing only 1% FBS and the phenotype confirmed by the absence of cell proliferation using absolute cell counts or absence of EdU (Thermo Fisher) incorporation. Senescence was induced either by transducing cells with lentiviral particles carrying the H‐RAS^V12^ oncogene or by exposure to ionizing irradiation (IR) using a Faxitron X‐Ray CP‐160. Doses ranged from 7 Gy for i‐LPC, 8 Gy for i‐EC, 12 Gy for myoblasts and 15 Gy for HDF/i‐HDF. Where indicated, cells were mock transduced using lentiviral particles carrying only the puromycin selection gene. For assays using expanded T cells, HDF were transduced with lentiviral particles carrying an inducible version of the K‐RAS‐ER oncogene (Singh et al. 2012) and senescence was induced by adding 100 nM of 4‐Hydroxytamoxifen (4‐HT) every 3 days. All cells were transduced overnight except for i‐LPC which were transduced over a 5‐h period in a single‐cell suspension containing 10 μM ROCK inhibitor. Unless indicated otherwise, senescence was assessed 10 days after treatments by evaluating cell morphology and the absence of proliferation. Growth curves were determined using an Incucyte S3 (Sartorius) or by counting the population doubling (PD) at each passage. Cells were stained with the Nuclight rapid dye (Sartorius) at a dilution of 1:750 and pictures were taken every 2 h over a period of up to 7 days. Medium and dye were replenished approximately every 3 days. Proliferation was assessed by the number of red nuclei per well over time. Senescence associated beta‐galactosidase (SA‐β‐gal) expression was confirmed using the previously described protocol by Itahana et al. (Itahana et al. 2013). Pictures were taken using the EVOS M5000 Imaging System and cell counts were performed using ImageJ on five different pictures taken from randomly selected areas within the well.

### 4.3 RNA Extraction and RT‐qPCR

Cells were plated at 3 × 10^5^ cells per 100 mm^3^ petri dish for each cell types. Senescence was induced and RNA harvested 10 days later. In brief, petri dishes were kept on ice and washed with cold PBS 3 times before adding 300 uL of Qiagen RLT buffer (catalog number 79216) + 1% b‐mercaptoethanol. Cells were then scraped and cell lysate transferred into a 1.5 mL Eppendorf for RNA extraction. Qiagen RNAeasy Mini kit (catalog number 74106) was used to extract RNA from cell lysate. RNA purity and quantity were assessed using a Nanodrop (ThermoFischer). To obtain cDNA, 1 μg of each sample was reverse transcribed (RT) using the Qiagen QuantiTect Reverse Transcription Kit (catalog number 205311). After RT, qPCR was done by using 1 μg cDNA of each sample using the PowerUp SYBR Green Master Mix (Thermofischer, 100,029,284). Primers used for hp16 were: (Forward: 5′‐CCAACGCACCGAATAGTTACG‐3′, Reverse: 5′‐GCGCTGCCCATCATCATG‐3′), hp21 (Forward: 5′GGCAGACCAGCATGACAGATT3′, Reverse: 5′‐GCGGATTAGGGCTTCCTCTT‐3′) and 18S (Forward: 5′‐CGCCGCTAGAGGTGAAATTCT‐3′, Reverse: 5′‐CGAACCTCCGACTTTCGTTCT‐3′).

### 4.4 Mass Spectrometry

HDF‐hTERT cells were expanded and induced to senesce by exposure to ionizing radiation (IR) at doses of 15 Gy or lentiviral particles carrying the H‐RASV12 oncogene. Cells were harvested 10 days after senescence induction. A total of 10^8^ cells per condition were collected by trypsinization, pelleted by centrifugation, and frozen at −80°C until further processing. MHC‐I: antigen complexes were purified by lysing cells in 1% CHAPS buffer in PBS containing protease inhibitors, followed by immunoprecipitation of the complexes as described previously (Kovalchik et al. 2022; Sirois et al. 2021). Tandem mass spectrometry (MS/MS) was performed using the Orbitrap Eclipse system. Identified peptides, including their predicted binding affinity to HLA molecules, were analyzed using NetMHCpan 4.0. Peptides were sorted and compared to the human HLA ligand atlas using Microsoft Excel and the interactive Venn diagram viewer jvenn. An in silico investigation of the predictive immunogenicity of senescence‐specific MHC‐I‐associated peptides (senMAPs) was performed using RNAseq data from human mTEC samples and other human tissues as previously described (Apavaloaei et al. 2022).

### 4.5 Immune Cells Isolation

Human adult peripheral blood was collected from healthy donors after informed consent, and immune cells were separated using a Ficoll‐Paque gradient (Cytiva). PBMCs were recovered from the buffy coat and washed twice with PBS. Granulocytes were isolated from the gradient pellet after Ficoll‐Paque removal. The cell pellet was dissociated and resuspended in sterile deionized water for 20 s to lyse red blood cells, followed by the addition of sterile 20X PBS solution.

For T cell isolation, PBMCs were resuspended in 40 μL of MACS buffer (PBS containing 0.5% BSA and 2 mM EDTA) per 10^7^ total cells, and 10 μL of a Pan T Cell Biotin‐Antibody Cocktail (Pan T Cell Isolation Kit, Human, Miltenyi, 130‐096‐535) was added. The cells were incubated in a refrigerator (2°C–7°C) for 5 min. After incubation, 30 μL of MACS buffer and 20 μL of Pan T Cell Microbead Cocktail were added per 10^7^ total cells, and the suspension was returned to the refrigerator for an additional 10 min. Magnetic cell separation was performed using an LS column in the magnetic field of a MACS separator. A 30 μm pre‐filter was applied before adding the cell suspension to the separation column. This procedure resulted in two fractions: one enriched in T cells (unlabeled) and the other containing non‐T cells. Both fractions were washed with PBS and subjected to a quick centrifugation.

NK cells were isolated from the buffy coat using the EasySep Human NK Cell Enrichment Kit (Stemcell Technologies), achieving an enrichment of 85% or more based on CD3^−^CD56^+^ expression, as determined by FACS. NK cells were either used fresh or incubated at 37°C with 5% CO_2_ in RPMI 1640 medium supplemented with 10% FBS, 1% penicillin/streptomycin (Multicell), and 100 U/mL IL‐2 (SteriMax) for 1–3 days. Otherwise, primed NK cells from the CD3^−^ cell fraction obtained from the EasySep Human CD3 Positive Selection Kit II (Stemcell Technologies) were expanded by coculture with irradiated (100 Gy) K562 cells expressing mbIL‐21 and 4‐1BBL. The coculture was maintained for a minimum of 2 weeks until the NK cell population (CD3^−^, CD56^+^) reached nearly 100%, as confirmed by FACS analysis. Expanded cells were rested for at least 7 days before being used in cytotoxicity assays.

### 4.6 FEST‐Assay

PBMCs were collected from the same donor of HDF‐hTERT used for the identification of senMAPs. PBMCs were separated into T cells and non‐T cells as explained above and the non‐T cell fraction was irradiated at 30 Gy. Non‐T cells and T cells were co‐cultured at a 1:1 ratio (10^6^ cells/mL) in a 6‐well tissue culture plate (TC‐Plate 6 well, SARSTEDT, 83.3920) using AIM‐V medium (Gibco) supplemented with 1% Hepes (Hepes 1 M solution, Multicell 330–050‐EL) and 50 μg/mL gentamicin (Gentamicin solution, G1397, Sigma). Peptides (from GL Biochem Shanghai) were added to the respective wells at a concentration of 1 μg/mL. Plates were incubated in a CO_2_ incubator at 37°C. On Days 3 and 7, cells were washed with PBS, and the medium was replaced. IL‐2 (100 IU/mL), IL‐15 (50 ng/mL), and IL‐7 (50 ng/mL) were added to the wells. On Day 10, a fresh fraction of non‐T cells was irradiated at 30 Gy. The freshly irradiated non‐T cells were incubated with the respective peptides at 37°C for 2 h, washed, and then added to the T cells at a 1:1 ratio. On Days 13 and 17, the medium was refreshed as described for Days 3 and 7. On Day 20, T cells were washed and rested in AIM‐V medium for 5 days before performing assays.

### 4.7 Elispot Assay

Millipore Multiscreen sterile IP plates with a high‐binding membrane (Millipore, cat. no. MSIPS4W10) were prepared by briefly incubating each well with 50 μL of 35% ethanol for less than 1 min. The plates were then washed three times with PBS. Following this, 100 μL per well of capture antibody solution (10 μg/mL purified mouse anti‐human IFN‐γ antibody, BD Pharmingen) was added, and the plates were incubated overnight at 4°C. After overnight incubation, the plates were washed five times with PBS. Each well was then filled with 200 μL of R10 medium and incubated at 37°C for 2 h. The plates were washed again five times with PBS before proceeding. Cells were added to the prepared plates and incubated for 22 h. Control wells consisted of T cells stimulated with 10 μL of a 1:50 dilution of anti‐CD3 (purified NA/LE mouse anti‐human CD3, BD Pharmingen). After the 22‐h incubation, cells were removed by inverting the plate, and the plates were washed five times with PBS‐Tween (PBS containing 0.05% Tween‐20) followed by five washes with PBS. A detection antibody solution (biotinylated mouse anti‐human IFN‐γ, BD Pharmingen) was prepared at a 1:1000 dilution in PBS containing 0.5% BSA. A volume of 100 μL of this solution was added to each well, and the plates were incubated at room temperature for 2 h, protected from light. After the 2‐h incubation, the plates were washed six times with PBS‐T and then twice with PBS. Streptavidin‐alkaline phosphatase (Sav‐AP, 1:1000 in PBS with 0.5% BSA) was added at 100 μL per well, and the plates were incubated at room temperature for 1 h, protected from light. The plates were washed three times with PBS‐T, followed by three washes with PBS. A developing solution was prepared using the AP buffer, Solution A, and Solution B from the AP conjugate substrate kit (Bio‐Rad, cat. no. 1706432) according to the manufacturer's instructions. A volume of 100 μL of the developing solution was added to each well. After a 5‐min incubation, the plates were washed thoroughly with tap water for 2 min per side. The plates were then left to dry overnight before analysis.

### 4.8 FACS Staining

Cells were resuspended in 100 μL of FACS buffer, consisting of PBS supplemented with 2% FBS. For surface marker detection, fluorochrome‐conjugated antibodies (as listed in Table S3) were added at the specified concentrations. To confirm specificity, isotype control antibodies were included at equivalent concentrations. The cell suspensions were incubated with antibodies for 15 min at 4°C in the dark. To evaluate cell viability, Zombie NIR (BioLegend) was used prior to antibody staining, following the manufacturer's protocol. After staining, cells were washed twice with FACS buffer and centrifuged at 400 × *g* for 5 min. In certain cases, cells were fixed with 4% paraformaldehyde (PFA) at room temperature for 10 min. Flow cytometry was performed on a BD LSRFortessa flow cytometer, and the resulting data were analyzed using FlowJo software.

### 4.9 In Vitro Cytotoxicity Assay

Target cells (5 × 10^4^ cells per well unless indicated otherwise) were seeded in 96‐well plates at least 2 days before the cytotoxicity assays. For i‐LPC, plates were coated with diluted Matrigel at a 1:1 ratio with DMEM. i‐LPC were seeded immediately after lentiviral transduction (H‐RASV12) or 4 days prior to exposure to IR. On the day of co‐culture, effector‐to‐target cell ratios were adjusted by plating the corresponding amount of immune cells. For co‐cultures with T cells, AIM‐V medium was used after a media change. PBMCs were added to target cells pretreated with 200 ng/mL IFN‐γ for 24 h, in the presence of 100 IU/mL IL‐2 and 0.25 μg/mL anti‐CD28 antibody. NK cells were co‐cultured in the presence of 20 IU/mL IL‐2. K562 cells were included as a positive control for NK cell‐mediated cytotoxicity. Target cell death was assessed 24 h later using flow cytometry. After staining with Zombie Aqua or NIR790 dye for viability, cells were detached with trypsin, resuspended in PBS containing 10% FBS, and analyzed on a FACS Fortessa High Throughput Sampler. T cell activation was determined by measuring CD69 expression on CD3^+^ cells, with anti‐CD3 antibody (OKT3) or Dynabeads serving as positive controls. Data were analyzed using FlowJo software. Experiments were conducted in triplicates, and the percentage of cell viability was calculated by comparing the average cell count in wells co‐cultured with immune cells to that in wells containing target cells alone.

### 4.10 Immunofluorescence

Desmin was detected in myoblasts that were plated at 50%–70% confluence on Geltrex‐coated coverslips and cultured for at least 2 days. Cells were fixed and permeabilized with 95% ethanol for 15 min, followed by blocking in PBS containing 10% FBS for 30 min with washes between steps. The anti‐human Desmin antibody was used at a dilution of 1:50 and incubated for 1 h. For the detection of Myosin heavy chain (MHC), myoblasts were plated at near confluence (80%–90%) and starved the following day in DMEM with 2% FBS for 5 days to promote myotube formation. Cells were fixed with 4% paraformaldehyde (PFA) for 15 min, washed, permeabilized with PBS containing 3% Triton X‐100, and incubated with an anti‐MHC antibody at 1:100 for 2 h. For von Willebrand factor (vWF), endothelial cells were fixed with 4% PFA, permeabilized, and blocked with PBS containing 10% FBS before overnight incubation with an anti‐vWF antibody at a dilution of 1:200 at 4°C. The detection of SPC/NKX2‐1 was performed in LPCs plated in Matrigel and cultured for 3 days to allow alveolosphere formation. Cells were fixed with 4% PFA for 15 min, blocked and permeabilized with PBS containing 0.1% Triton X‐100 and 1% BSA for 1 h at 37°C, and incubated overnight at 4°C with primary antibodies. Matrigel was re‐solidified by transferring cells to 37°C for 30 min. For CD4 and CD8 detection in mouse tibialis sections, cryosections of 12 μm thickness were mounted on slides treated with gelatin and chromo alum. Sections were fixed with 4% PFA for 15 min, washed, and blocked with PBS containing 10% FBS and 0.3 M glycine for 30 min at room temperature. Primary antibodies diluted 1:200 in blocking buffer were applied overnight at 4°C under paraffin film. In all experiments, secondary antibody staining was performed using Alexa 594 (1:500, Invitrogen) for 30 min. DAPI staining was done at 0.5 μg/mL, and samples were mounted with Vectashield. Imaging was conducted using an Olympus BX51 microscope equipped with a Qimaging Retiga 2000R camera. For CD4 and CD8 staining, human immune cell infiltration was quantified by averaging the counts from five areas with the highest cell density per slide.

### 4.11 Quantification of SASP Factors

For the quantification of SASP factors, conditioned media was collected as described in (Goyer et al. 2024). In brief, each cell line was incubated for 24 h in their specific medium in the absence of FBS. After the incubation period, the samples were sent to Eve Technologies Corporation (AB, Canada) for analysis. The cytokine profiles were assessed using the Human Cytokine Panel A 48‐Plex or the 65‐Plex. The results are represented as the log10 transformation of the normalized concentration after scaling the data on a common denominator.

### 4.12 Endothelial Cell Tube Formation Assay (TFA)

Endothelial cells were resuspended in LVES supplemented medium (Gibco, M200500, A1460801) and plated at a concentration of 5 × 10^4^ per well in a 12 well plate previously coated with 200μL of undiluted Geltrex. Cells were incubated at 37°C, 5% O_2_ for 5 h before pictures were taken with the EVOS XL Core Imaging System.

### 4.13 Fetal Myoblast Transplantation and Monitoring in BLT Humanized Mice

Bone marrow, liver, and thymus humanized mice (BLT‐hu) were generated by surgically implanting fetal liver and thymus fragments (1–2 mm^3^) under the renal capsule and injecting intravenously autologous CD34+ hematopoietic stem cells collected from the fetal liver in 6–8‐week‐old NOD/SCID/IL2Rγ null (NSG) mice previously exposed to 2 Gy of total body irradiation using a Faxitron CP‐160. The fetal tissues (16–21 weeks) were obtained with written informed consent (ethical committee of CHU Sainte‐Justine, CER#2126). Hematopoietic engraftment was evaluated by flow cytometry from peripheral blood (PB) at 8 and 13 weeks post‐surgery, and again at the time of sacrifice. At week 14, 1 × 106 autologous luciferase‐expressing fetal myoblasts (fMB), suspended in 20 μL of PBS with 10 μg/mL of cardiotoxin (Sigma, 217,503), were transplanted into multiple sites of the tibialis anterior (TA) muscle of the mice through approximately 20 percutaneous microinjections. The luciferase signal was monitored over 3 weeks using the Q‐Lumi In Vivo imaging system (MediLumine, Montreal). Images were standardized internally and normalized using FIJI macros for picture processing. After sacrifice, TA muscles were collected, embedded in OCT compound (VWR), and flash frozen in liquid nitrogen. The spleen was mechanically crushed and digested with dispase (Stemcell Technologies) and collagenase (Roche), and human immune cell quantification was performed by FACS.

### 4.14 I‐LPC Injection and Monitoring in Mice

To assess the engraftment and clearance of i‐LPC, 7.5 × 10^5^ (Mock and H‐RAS) or 1 × 10^6^ (Ctrl and IR) i‐LPC were stained with 2 μM Biotracker NIR790 cytoplasmic membrane dye (EMD Millipore) and injected intravenously in 100 μL of RPMI1640 containing 10 μM of Rock inhibitor into NSG‐SGM3 mice. Six hours after i‐LPC injection, PBMCs and granulocytes (5 × 10^6^ each) were injected intraperitoneally in 200 μL of RPMI1640. The NIR790 fluorescence signal from i‐LPC was monitored twice a week for 17 days using the Q‐Lumi In Vivo imaging system, with an excitation wavelength of 769–41 nm and an emission wavelength of 832–37 nm. Fluorescence signals were standardized internally for each image and processed using ImageJ macros for normalization. The corrected total organ fluorescence (CTOF) was calculated by adjusting for background fluorescence and area, providing a quantitative measure of i‐LPC distribution and trafficking in the mice.

### 4.15 Statistics

Graphic design and statistical analyses were conducted using GraphPad Prism version 9.3.1 unless otherwise stated. Significance level was set at *α* = 0.05.

## Author Contributions

M‐.L.G., A.S., J.D., O.L., J.O., I.S., A.A., B.B., performed experiments. M‐.L.G., A.S. and C.B. designed the studies. E.H., L.H., J.V.G. provided reagents and expertise. M‐.L.G., A.S. and C.B. wrote the manuscript.

## Funding

This research was funded by a grant from the Natural Sciences and Engineering Research Council of Canada (RGPIN‐2023‐03683) and by the Terry Fox new frontiers program project grants to C.B. M‐.L.G. was supported by a studentship from the Fonds de recherche du Québec en Santé (FRQS). J.D. was supported in part by a post‐doctoral fellowship from the Cole Foundation.

## Conflicts of Interest

The authors declare no conflicts of interest.

## Supporting information


**Figure S1:** acel70410‐sup‐0001‐FigureS1.pdf.

**Table S1:** acel70410‐sup‐0002‐TableS2.docx.

## Data Availability

The data that supports the findings of this study are in part available in the supporting information S1 of this article and available from the corresponding author upon reasonable request.

## References

[acel70410-bib-0001] Apavaloaei, A. , L. Hesnard , M. P. Hardy , et al. 2022. “Induced Pluripotent Stem Cells Display a Distinct Set of MHC I‐Associated Peptides Shared by Human Cancers.” Cell Reports 40, no. 7: 111241. 35977509 10.1016/j.celrep.2022.111241

[acel70410-bib-0002] Armenian, S. H. , C. J. Gibson , R. C. Rockne , and K. K. Ness . 2019. “Premature Aging in Young Cancer Survivors.” Journal of the National Cancer Institute 111, no. 3: 226–232. 30715446 10.1093/jnci/djy229

[acel70410-bib-0003] Baker, D. J. , B. G. Childs , M. Durik , et al. 2016. “Naturally Occurring p16(Ink4a)‐Positive Cells Shorten Healthy Lifespan.” Nature 530, no. 7589: 184–189. 26840489 10.1038/nature16932 PMC4845101

[acel70410-bib-0004] Beausejour, C. M. , A. Krtolica , F. Galimi , et al. 2003. “Reversal of Human Cellular Senescence: Roles of the p53 and p16 Pathways.” EMBO Journal 22, no. 16: 4212–4222. 12912919 10.1093/emboj/cdg417 PMC175806

[acel70410-bib-0005] Benabdallah, B. , C. Désaulniers‐Langevin , C. Colas , et al. 2019. “Natural Killer Cells Prevent the Formation of Teratomas Derived From Human Induced Pluripotent Stem Cells.” Frontiers in Immunology 10: 2580. 31787975 10.3389/fimmu.2019.02580 PMC6854018

[acel70410-bib-0006] Benabdallah, B. , C. Désaulniers‐Langevin , M. L. Goyer , et al. 2021. “Myogenic Progenitor Cells Derived From Human Induced Pluripotent Stem Cell Are Immune‐Tolerated in Humanized Mice.” Stem Cells Translational Medicine 10, no. 2: 267–277. 32881406 10.1002/sctm.19-0452 PMC7848353

[acel70410-bib-0007] Brighton, P. J. , Y. Maruyama , K. Fishwick , et al. 2017. “Clearance of Senescent Decidual Cells by Uterine Natural Killer Cells in Cycling Human Endometrium.” eLife 6: 6. 10.7554/eLife.31274 PMC5724991 29227245

[acel70410-bib-0008] Caron, E. , K. Vincent , M.‐. H. Fortier , et al. 2011. “The MHC I Immunopeptidome Conveys to the Cell Surface an Integrative View of Cellular Regulation.” Molecular Systems Biology 7: 533. 21952136 10.1038/msb.2011.68 PMC3202804

[acel70410-bib-0009] Chaib, S. , J. A. López‐Domínguez , M. Lalinde‐Gutiérrez , et al. 2024. “The Efficacy of Chemotherapy Is Limited by Intratumoral Senescent Cells Expressing PD‐L2.” Nature Cancer 5, no. 3: 448–462. 38267628 10.1038/s43018-023-00712-x PMC10965441

[acel70410-bib-0010] Childs, B. G. , D. J. Baker , T. Wijshake , C. A. Conover , J. Campisi , and J. van Deursen . 2016. “Senescent Intimal Foam Cells Are Deleterious at All Stages of Atherosclerosis.” Science 354, no. 6311: 472–477. 27789842 10.1126/science.aaf6659 PMC5112585

[acel70410-bib-0011] Chuprin, J. , H. Buettner , M. O. Seedhom , et al. 2023. “Humanized Mouse Models for Immuno‐Oncology Research.” Nature Reviews. Clinical Oncology 20, no. 3: 192–206. 10.1038/s41571-022-00721-2 PMC10593256 36635480

[acel70410-bib-0012] Conte, T. C. , G. Duran‐Bishop , Z. Orfi , et al. 2023. “Clearance of Defective Muscle Stem Cells by Senolytics Restores Myogenesis in Myotonic Dystrophy Type 1.” Nature Communications 14, no. 1: 4033. 10.1038/s41467-023-39663-3 PMC10356779 37468473

[acel70410-bib-0013] Cruz‐Barrera, M. , J. Dulong , G. Mansour Nehmo , et al. 2025. “Senescent Human Fibroblasts Have Increased FasL Expression and Impair the Tumor Immune Response.” Frontiers in Immunology 16: 1685269. 41221280 10.3389/fimmu.2025.1685269 PMC12597918

[acel70410-bib-0014] Egashira, M. , Y. Hirota , R. Shimizu‐Hirota , et al. 2017. “F4/80+ Macrophages Contribute to Clearance of Senescent Cells in the Mouse Postpartum Uterus.” Endocrinology 158, no. 7: 2344–2353. 28525591 10.1210/en.2016-1886

[acel70410-bib-0015] Faget, D. V. , Q. Ren , and S. A. Stewart . 2019. “Unmasking Senescence: Context‐Dependent Effects of SASP in Cancer.” Nature Reviews Cancer 19, no. 8: 439–453. 31235879 10.1038/s41568-019-0156-2

[acel70410-bib-0016] Goyer, M. L. , C. Desaulniers‐Langevin , A. Sonn , et al. 2024. “Induced Pluripotent Stem Cell‐Derived Fibroblasts Efficiently Engage Senescence Pathways but Show Increased Sensitivity to Stress Inducers.” Cells 13, no. 10: 849. 38786071 10.3390/cells13100849 PMC11119907

[acel70410-bib-0017] Hasegawa, T. , T. Oka , H. G. Son , et al. 2023. “Cytotoxic CD4(+) T Cells Eliminate Senescent Cells by Targeting Cytomegalovirus Antigen.” Cell 186, no. 7: 1417–1431.e20. 37001502 10.1016/j.cell.2023.02.033

[acel70410-bib-0018] Iannello, A. , T. W. Thompson , M. Ardolino , S. W. Lowe , and D. H. Raulet . 2013. “p53‐Dependent Chemokine Production by Senescent Tumor Cells Supports NKG2D‐Dependent Tumor Elimination by Natural Killer Cells.” Journal of Experimental Medicine 210, no. 10: 2057–2069. 24043758 10.1084/jem.20130783 PMC3782044

[acel70410-bib-0019] Iltis, C. , I. Moskalevska , A. Debiesse , et al. 2025. “A Ganglioside‐Based Immune Checkpoint Enables Senescent Cells to Evade Immunosurveillance During Aging.” Nature Aging 5, no. 2: 219–236. 39730825 10.1038/s43587-024-00776-z PMC11839482

[acel70410-bib-0020] Itahana, K. , Y. Itahana , and G. P. Dimri . 2013. “Colorimetric Detection of Senescence‐Associated β Galactosidase.” Methods in Molecular Biology 965: 143–156. 23296655 10.1007/978-1-62703-239-1_8 PMC3769963

[acel70410-bib-0021] Jacob, A. , M. Vedaie , D. A. Roberts , et al. 2019. “Derivation of Self‐Renewing Lung Alveolar Epithelial Type II Cells From Human Pluripotent Stem Cells.” Nature Protocols 14, no. 12: 3303–3332. 31732721 10.1038/s41596-019-0220-0 PMC7275645

[acel70410-bib-0022] Kang, T. W. , T. Yevsa , N. Woller , et al. 2011. “Senescence Surveillance of Pre‐Malignant Hepatocytes Limits Liver Cancer Development.” Nature 479, no. 7374: 547–551. 22080947 10.1038/nature10599

[acel70410-bib-0023] Katsuumi, G. , I. Shimizu , M. Suda , et al. 2024. “SGLT2 Inhibition Eliminates Senescent Cells and Alleviates Pathological Aging.” Nature Aging 4, no. 7: 926–938. 38816549 10.1038/s43587-024-00642-y PMC11257941

[acel70410-bib-0024] Kovalchik, K. A. , Q. Ma , L. Wessling , et al. 2022. “MhcVizPipe: A Quality Control Software for Rapid Assessment of Small‐ To Large‐Scale Immunopeptidome Datasets.” Molecular & Cellular Proteomics 21, no. 1: 100178. 34798331 10.1016/j.mcpro.2021.100178 PMC8717601

[acel70410-bib-0025] Le, O. N. , O. N. L. Le , F. Rodier , et al. 2010. “Ionizing Radiation‐Induced Long‐Term Expression of Senescence Markers in Mice Is Independent of p53 and Immune Status.” Aging Cell 9, no. 3: 398–409. 20331441 10.1111/j.1474-9726.2010.00567.x PMC2894262

[acel70410-bib-0026] Maggiorani, D. , O. le , V. Lisi , et al. 2024. “Senescence Drives Immunotherapy Resistance by Inducing an Immunosuppressive Tumor Microenvironment.” Nature Communications 15, no. 1: 2435. 10.1038/s41467-024-46769-9 PMC10948808 38499573

[acel70410-bib-0027] Majewska, J. , A. Agrawal , A. Mayo , et al. 2024. “p16‐Dependent Increase of PD‐L1 Stability Regulates Immunosurveillance of Senescent Cells.” Nature Cell Biology 26, no. 8: 1336–1345. 39103548 10.1038/s41556-024-01465-0 PMC11321988

[acel70410-bib-0028] Marcoux, S. , O. N. L. Le , C. Langlois‐Pelletier , et al. 2013. “Expression of the Senescence Marker p16INK4a in Skin Biopsies of Acute Lymphoblastic Leukemia Survivors: A Pilot Study.” Radiation Oncology 8: 252. 24171943 10.1186/1748-717X-8-252 PMC3827993

[acel70410-bib-0029] Marcu, A. , L. Bichmann , L. Kuchenbecker , et al. 2021. “HLA Ligand Atlas: A Benign Reference of HLA‐Presented Peptides to Improve T‐Cell‐Based Cancer Immunotherapy.” Journal for Immunotherapy of Cancer 9, no. 4: e002071. 33858848 10.1136/jitc-2020-002071 PMC8054196

[acel70410-bib-0030] Marin, I. , O. Boix , A. Garcia‐Garijo , et al. 2023. “Cellular Senescence Is Immunogenic and Promotes Antitumor Immunity.” Cancer Discovery 13, no. 2: 410–431. 36302218 10.1158/2159-8290.CD-22-0523 PMC7614152

[acel70410-bib-0031] Mason, J. A. , C. A. Davison‐Versagli , A. K. Leliaert , et al. 2016. “Oncogenic Ras Differentially Regulates Metabolism and Anoikis in Extracellular Matrix‐Detached Cells.” Cell Death and Differentiation 23, no. 8: 1271–1282. 26915296 10.1038/cdd.2016.15 PMC4947665

[acel70410-bib-0032] Michaloglou, C. , L. C. Vredeveld , M. S. Soengas , et al. 2005. “BRAFE600‐Associated Senescence‐Like Cell Cycle Arrest of Human Naevi.” Nature 436, no. 7051: 720–724. 16079850 10.1038/nature03890

[acel70410-bib-0033] Muñoz, D. P. , S. M. Yannone , A. Daemen , et al. 2019. “Targetable Mechanisms Driving Immunoevasion of Persistent Senescent Cells Link Chemotherapy‐Resistant Cancer to Aging.” JCI Insight 5, no. 14. 10.1172/jci.insight.124716 PMC6675550 31184599

[acel70410-bib-0034] Ovadya, Y. , T. Landsberger , H. Leins , et al. 2018. “Impaired Immune Surveillance Accelerates Accumulation of Senescent Cells and Aging.” Nature Communications 9, no. 1: 5435. 10.1038/s41467-018-07825-3 PMC6303397 30575733

[acel70410-bib-0035] Palacio, L. , M. L. Goyer , D. Maggiorani , et al. 2019. “Restored Immune Cell Functions Upon Clearance of Senescence in the Irradiated Splenic Environment.” Aging Cell 18, no. 4: e12971. 31148373 10.1111/acel.12971 PMC6612633

[acel70410-bib-0036] Pereira, B. , O. P. Devine , M. Vukmanovic‐Stejic , et al. 2019. “Senescent Cells Evade Immune Clearance via HLA‐E‐Mediated NK and CD8+ T Cell Inhibition.” Nature Communications 10, no. 1. 10.1038/s41467-019-10335-5 PMC6547655 31160572

[acel70410-bib-0037] Roos, C. M. , B. Zhang , A. K. Palmer , et al. 2016. “Chronic Senolytic Treatment Alleviates Established Vasomotor Dysfunction in Aged or Atherosclerotic Mice.” Aging Cell 15, no. 5: 973–977. 26864908 10.1111/acel.12458 PMC5013022

[acel70410-bib-0038] Ruscetti, M. , J. Leibold , M. J. Bott , et al. 2018. “NK Cell‐Mediated Cytotoxicity Contributes to Tumor Control by a Cytostatic Drug Combination.” Science 362, no. 6421: 1416–1422. 30573629 10.1126/science.aas9090 PMC6711172

[acel70410-bib-0039] Sagiv, A. , D. G. Burton , Z. Moshayev , et al. 2016. “NKG2D Ligands Mediate Immunosurveillance of Senescent Cells.” Aging (Albany NY) 8, no. 2: 328–344. 26878797 10.18632/aging.100897 PMC4789586

[acel70410-bib-0040] Sceneay, J. , G. J. Goreczny , K. Wilson , et al. 2019. “Interferon Signaling Is Diminished With Age and Is Associated With Immune Checkpoint Blockade Efficacy in Triple‐Negative Breast Cancer.” Cancer Discovery 9, no. 9: 1208–1227. 31217296 10.1158/2159-8290.CD-18-1454 PMC11167954

[acel70410-bib-0041] Singh, A. , M. F. Sweeney , M. Yu , et al. 2012. “TAK1 Inhibition Promotes Apoptosis in KRAS‐Dependent Colon Cancers.” Cell 148, no. 4: 639–650. 22341439 10.1016/j.cell.2011.12.033 PMC3291475

[acel70410-bib-0042] Sirois, I. , M. Isabelle , J. D. Duquette , F. Saab , and E. Caron . 2021. “Immunopeptidomics: Isolation of Mouse and Human MHC Class I‐ and II‐Associated Peptides for Mass Spectrometry Analysis.” Journal of Visualized Experiments 176. 10.3791/63052 34723952

[acel70410-bib-0043] Suryadevara, V. , A. D. Hudgins , A. Rajesh , et al. 2024. “SenNet Recommendations for Detecting Senescent Cells in Different Tissues.” Nature Reviews. Molecular Cell Biology 25, no. 12: 1001–1023. 38831121 10.1038/s41580-024-00738-8 PMC11578798

[acel70410-bib-0044] Tian, Y. , H. Li , T. Qiu , et al. 2019. “Loss of PTEN Induces Lung Fibrosis via Alveolar Epithelial Cell Senescence Depending on NF‐κB Activation.” Aging Cell 18, no. 1: e12858. 30548445 10.1111/acel.12858 PMC6351835

[acel70410-bib-0045] Wang, T. W. , Y. Johmura , N. Suzuki , et al. 2022. “Blocking PD‐L1‐PD‐1 Improves Senescence Surveillance and Ageing Phenotypes.” Nature 611, no. 7935: 358–364. 36323784 10.1038/s41586-022-05388-4

[acel70410-bib-0046] Xue, W. , L. Zender , C. Miething , et al. 2007. “Senescence and Tumour Clearance Is Triggered by p53 Restoration in Murine Liver Carcinomas.” Nature 445, no. 7128: 656–660. 17251933 10.1038/nature05529 PMC4601097

[acel70410-bib-0047] Yamada, Z. , J. Nishio , K. Motomura , et al. 2022. “Senescence of Alveolar Epithelial Cells Impacts Initiation and Chronic Phases of Murine Fibrosing Interstitial Lung Disease.” Frontiers in Immunology 13: 935114. 36059455 10.3389/fimmu.2022.935114 PMC9434111

