# Supplementary material for: The Immunogenicity of Human Senescent Cells Is Dependent on the Senescence Inducer and Cell Type
Source: Aging Cell. 2026 Feb 12;25(2):e70410. doi: 10.1111/acel.70410 (PMC12900895; doi:10.1111/acel.70410)
Supplement: Supplementary file 1 — Figure S1: acel70410‐sup‐0001‐FigureS1.pdf. [file ACEL-25-e70410-s002.pdf]

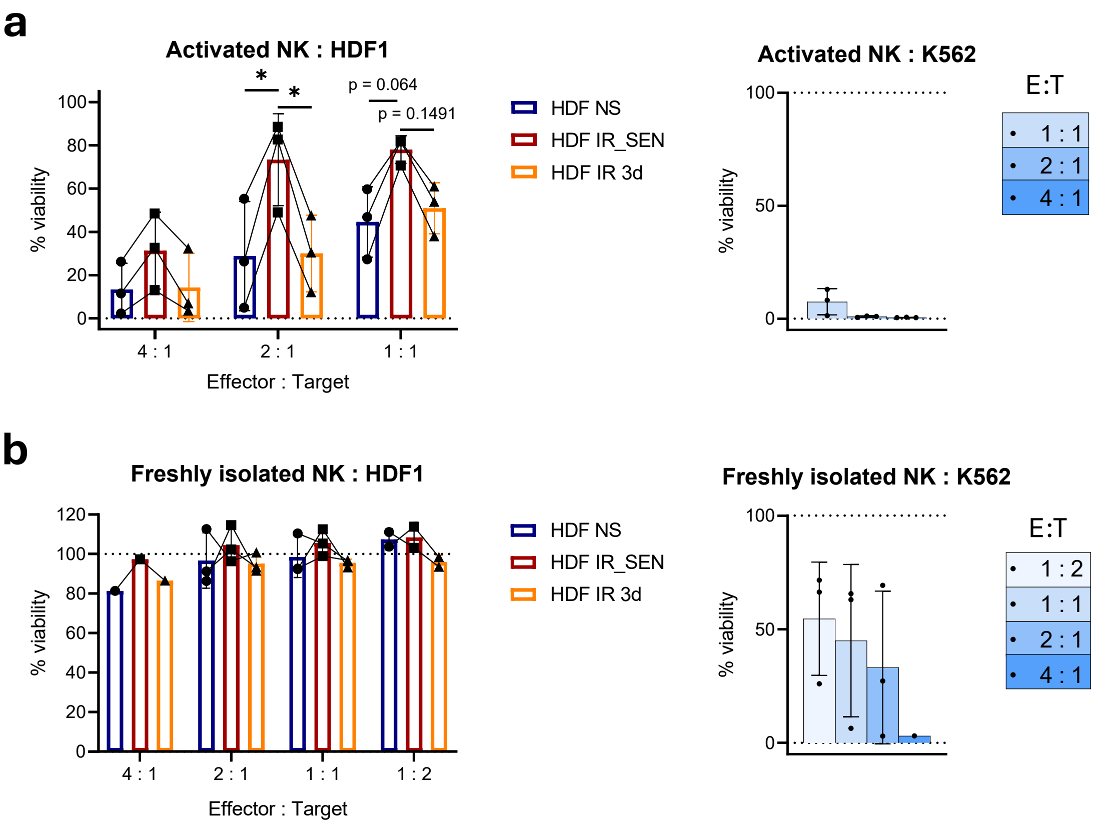

Figure S1. Senescent human dermal fibroblasts are protected from the cytotoxicity of activated NK cells when plated at high density. A-B) Shown is the viability of non-senescent (NS) HDF, HDF induced to senesce for 7 days following exposure to IR (IR_SEN) or induced to senesce for only 3 days (3 days post-IR). HDF were plated at high density (2x104 cells per well) and viability evaluated after 24 hours of coculture with activated NK cells (panel A) or freshly isolated NK cells (panel B). Cell counts were determined using flow cytometry. Shown is the mean ± SD of 3 different biological replicates performed with different NK cell donors, each connected by line. Viability of K562 serving as cytotoxicity positive control. Statistical analysis was performed using a two-way ANOVA followed by Tukey's multiple comparisons test. *: $p < 0.05$.

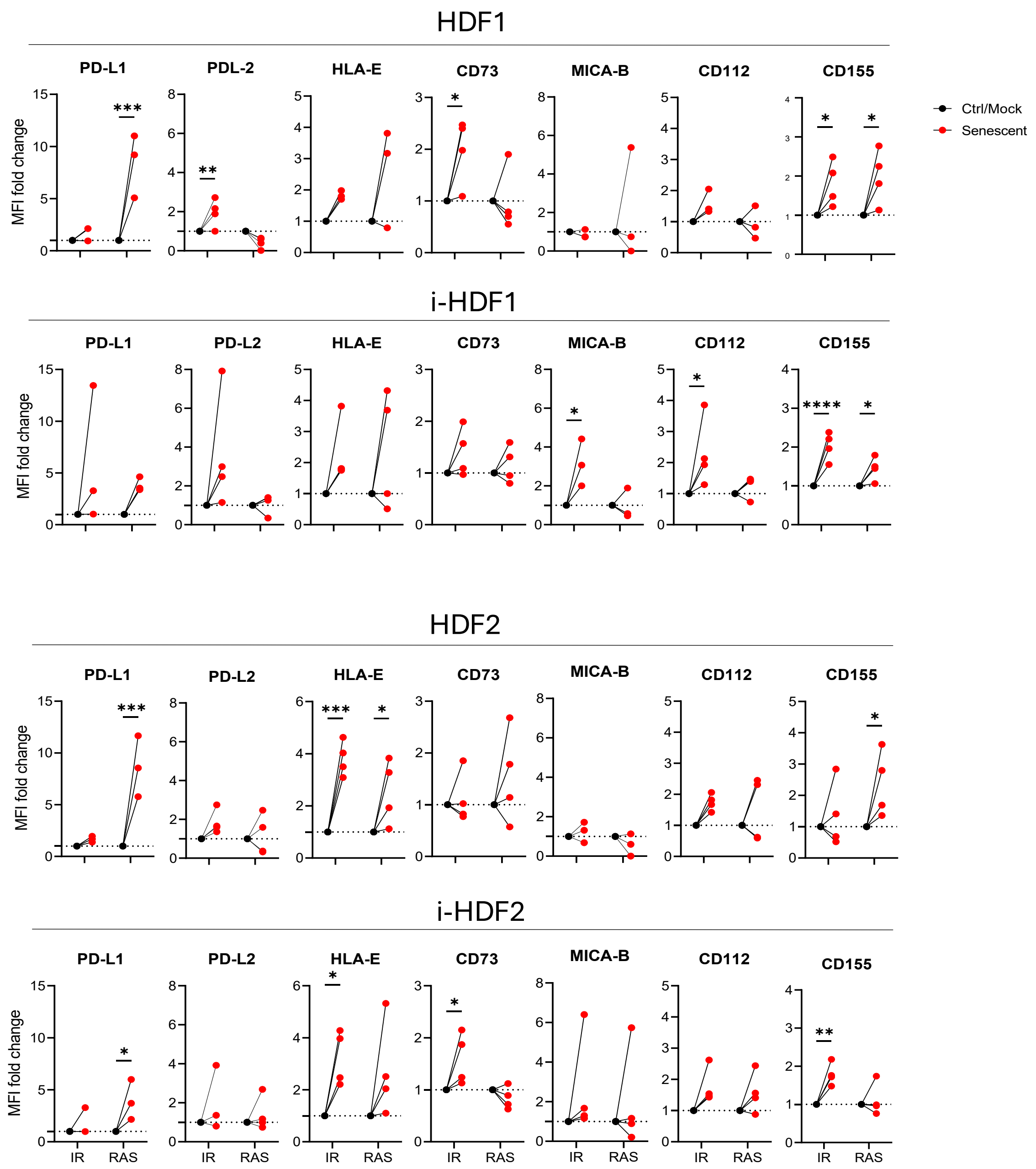

Figure S2. Expression of immune surface ligands in human fibroblasts. Mean fluorescence intensity (MFI) fold change as determined by flow cytometry of various immune surface ligands from senescent HDF and i-HDF compared to their respective non-senescent controls. Shown is the mean of n=3-4 independent biological experiments performed in triplicate. Statistical analysis was performed using a two-way ANOVA followed by Fisher's LSD test.

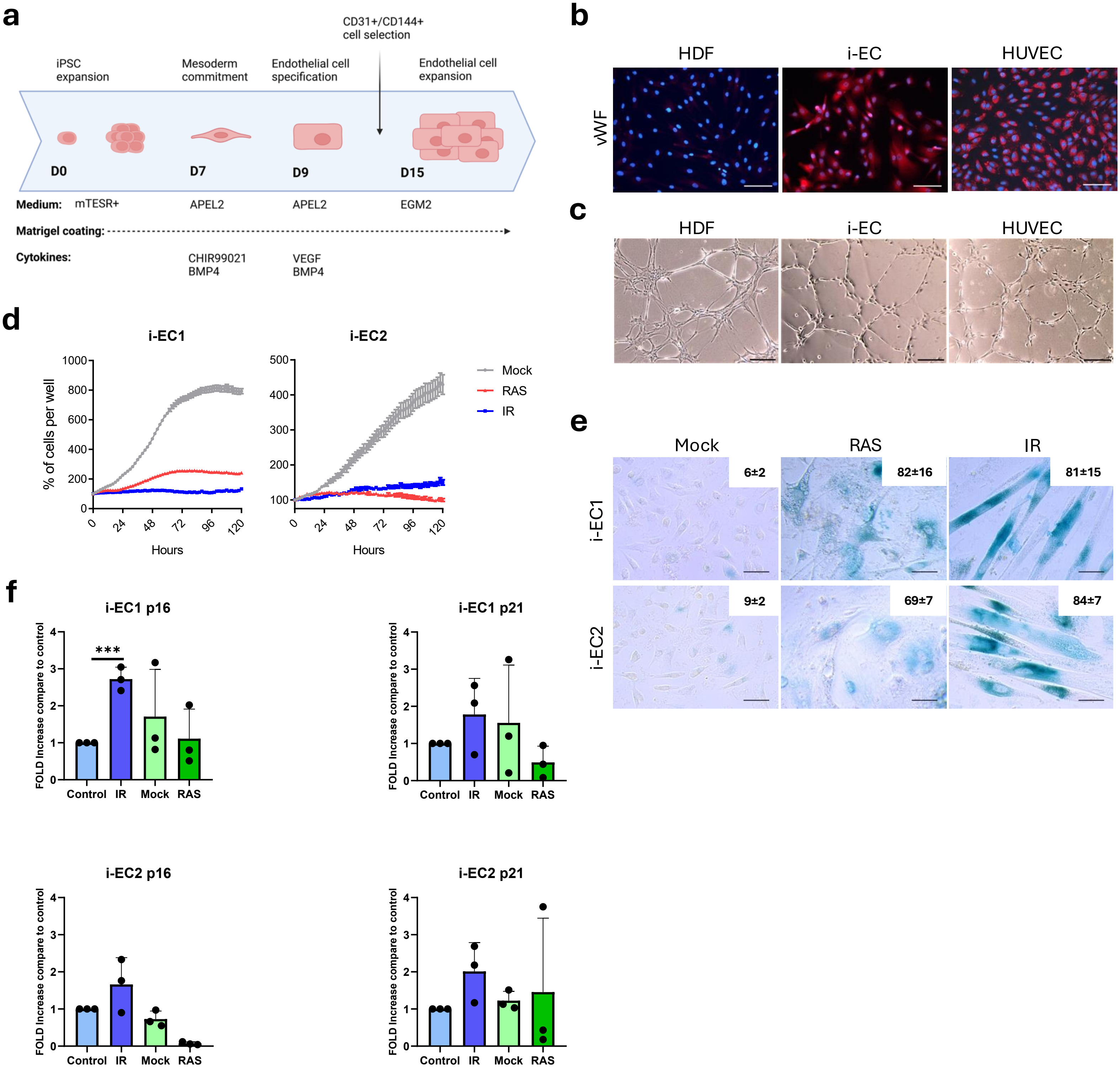

Figure S3. Differentiation and senescence induction of i-EC. A) Schematic timeline illustrating the differentiation of iPSC into i-EC. **B)** Immunofluorescence staining for von Willebrand factor (vWF) in i-EC, with HDF and HUVEC serving as negative and positive controls respectively. Scale bar = 100 uM. **C)** Representative images of tubule formation by i-EC cultured on Matrigel for 5 hours. Scale bar = 100 uM. **D)** Growth curve of i-EC expressing GFP nuclear staining from two donors following senescence induction, normalized to the initial time point using Incucyte. **E)** i-EC cells from both donors were stained for the SA-β-gal marker 10 days post senescence induction. Scale bar = 50 uM. The proportion of SA-β-gal positive cells is indicated on the top right corner. Cells were counted manually and the mean ± SD of three independent experiments is shown. **F)** Relative expression of p16 and p21 from 2 donors (i-EC1 and i-EC2) normalized to the control is shown. Mock cells were transduced with an empty lentivirus and serve as control for RAS-transduced cells. Shown is the mean ± SD from 3 biological replicates. Statistical analysis were performed by t-test ***$p < 0.001$.

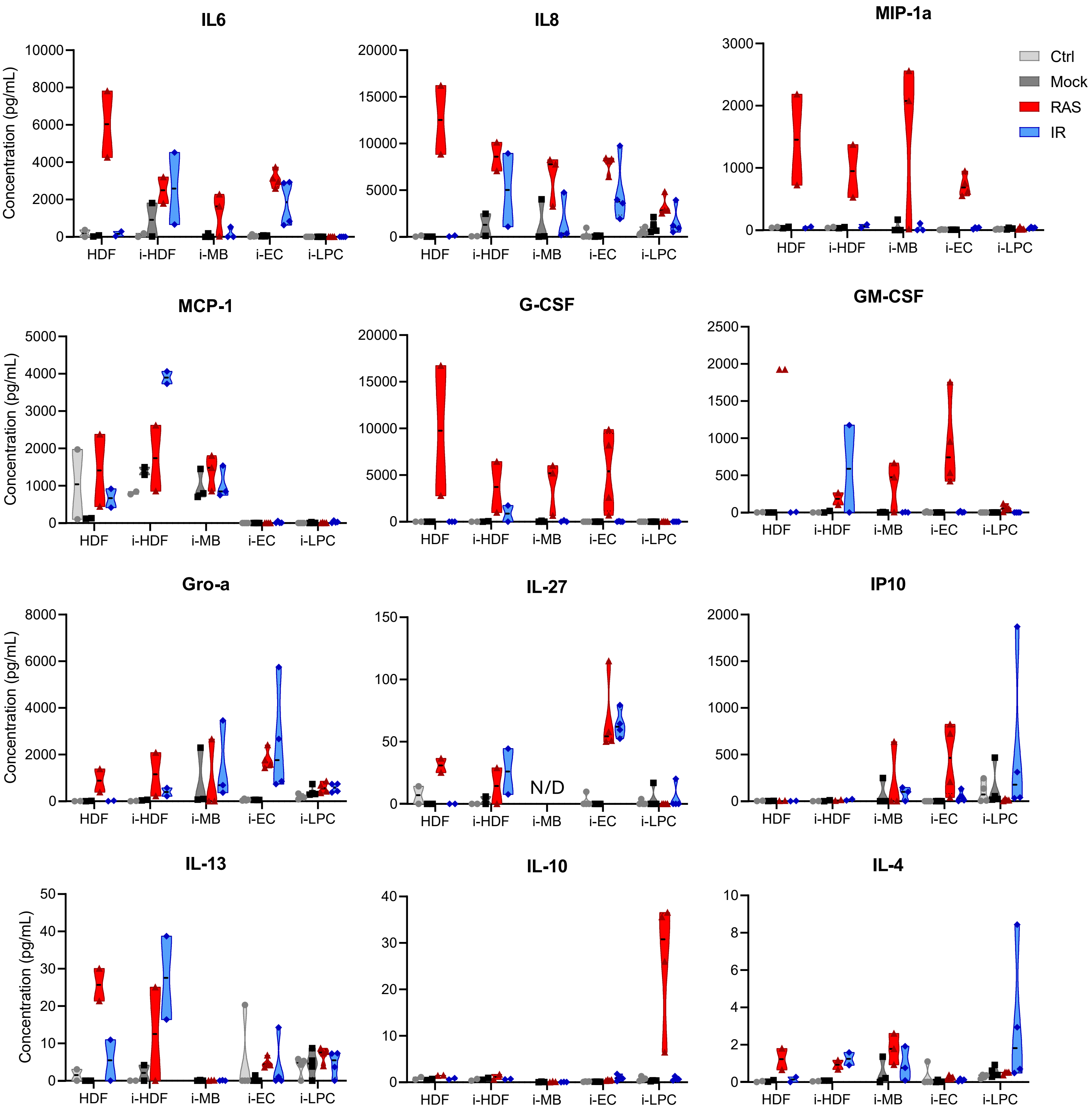

Figure S4. Distinct SASP profiles across cell types and senescence inducers. Concentration of the most abundant cytokines or those displaying significant variation between the different indicated cell types and senescence inducers. Undetected values were corrected to zero. Shown is the mean ± SD from 3-4 biological replicates. N/D: Not determined.

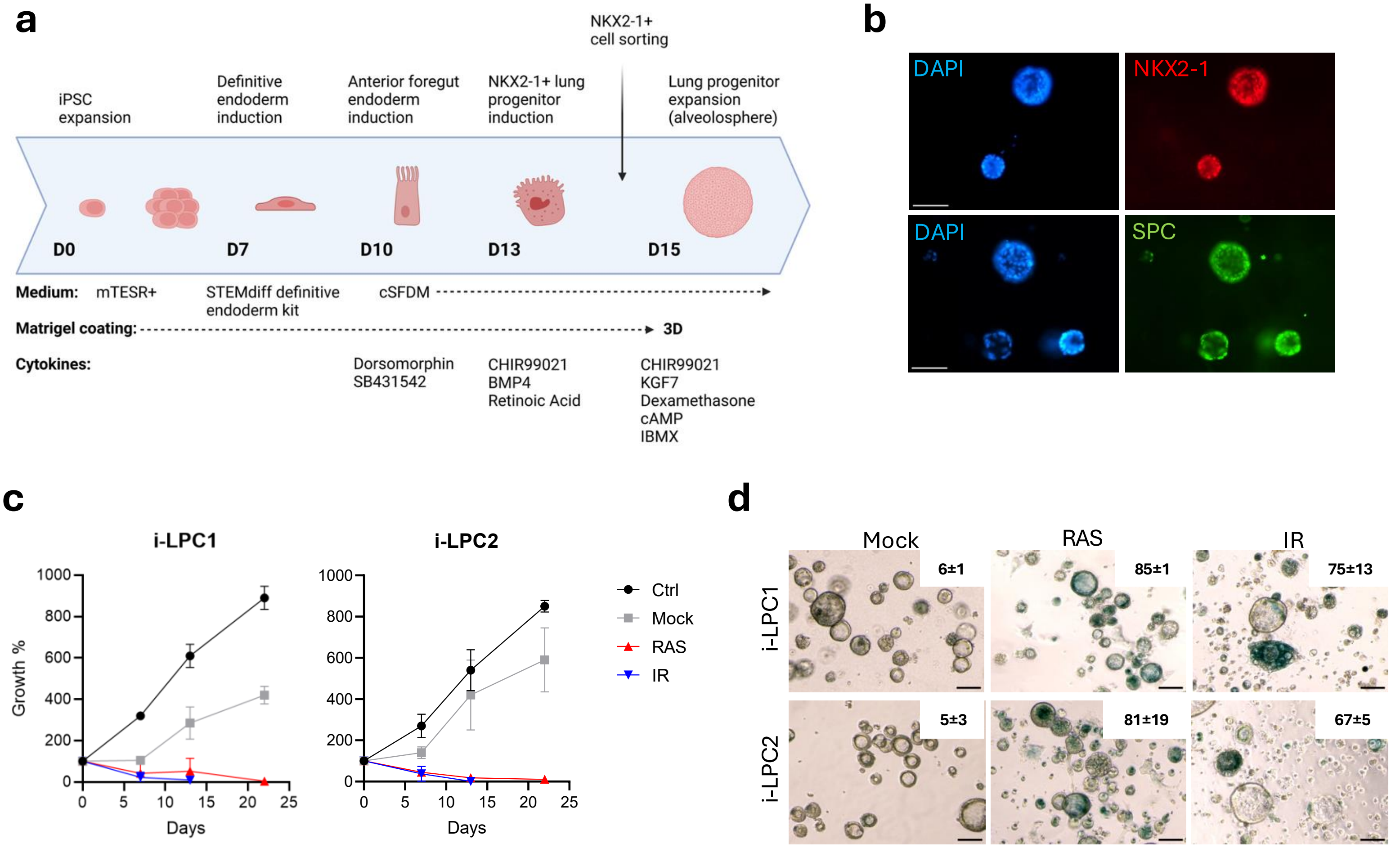

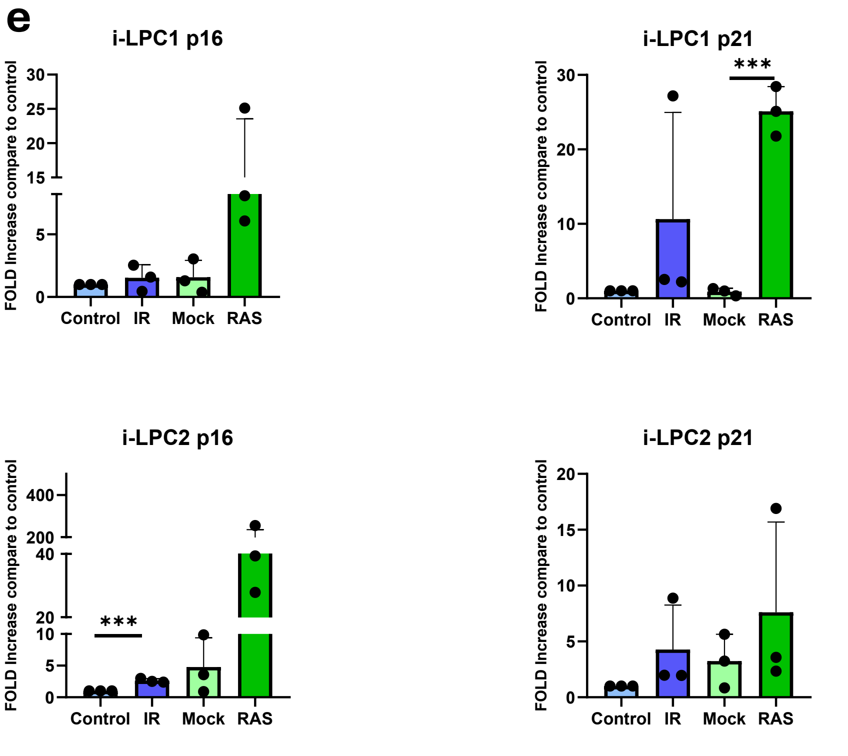

Figure S5. Differentiation and senescence induction of i-LPC. A) Schematic timeline illustrating the differentiation of iPSC into i-LPC. **B)** Immunofluorescence staining for NKX2-1 and SPC on i-LPC. Scale bar = 100 uM. **C)** Shown is the proliferation of i-LPC from two donors following exposure or not to senescence inducers. Spheres were dissociated for counting individual cells. **D)** i-LPC spheroids stained for the SA-β- gal marker 10 days post senescence induction. Scale bar = 100 uM. The proportion of SA-β-gal positive cells is indicated on the top right corner. Cells were counted manually and the mean ± SD of three independent experiments is shown. SPC: Surfactant Protein C. **E)** Relative expression of p16 and p21 from 2 donors (i-LPC1 and i-LPC2) normalized to the control is shown. Mock cells were transduced with an empty lentivirus and serve as control for RAS-transduced cells. Shown is the mean ± SD from 3 biological replicates. Statistical analysis were performed by t-test. ***p < 0.001.

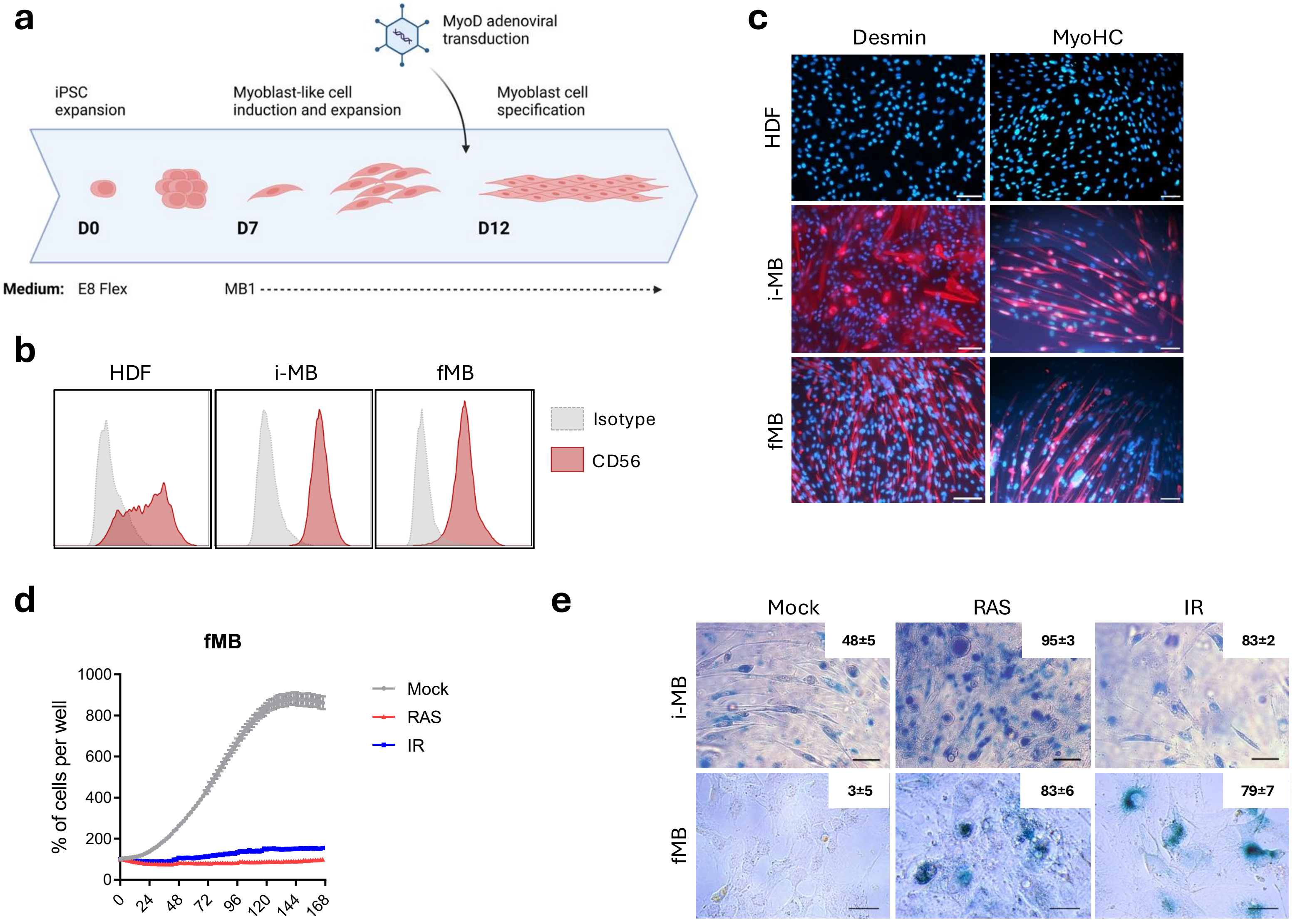

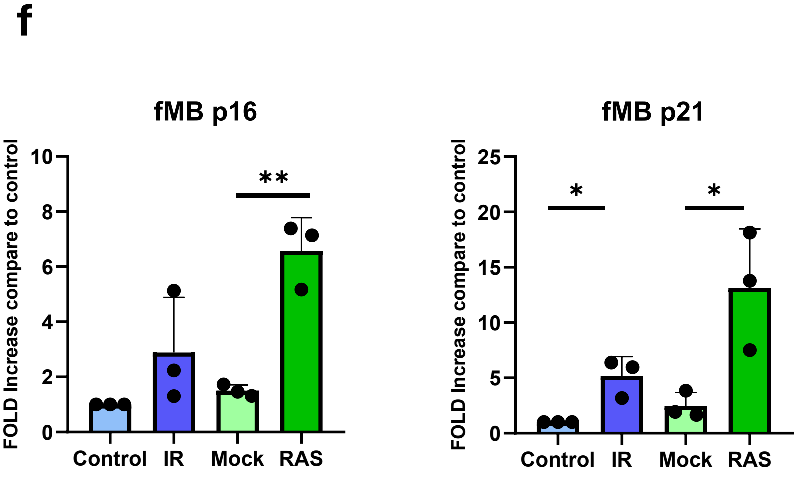

Figure S6. Differentiation of i-MB and senescence induction of i-MB and fMB. A) Schematic timeline illustrating the differentiation of iPSC into i-MB. **B)** Proportion of i-MB and fMB expressing CD56 as determined by flow cytometry. HDF were used as negative control. **C)** Immunofluorescence staining for desmin and MyoHC on i-MB and fMB. HDF were used as a negative control. Scale bar = 100 uM. **D)** Growth curve of fMB following senescence induction as determined using Incucyte. **E)** i-MB and fMB stained for the SA-β-gal marker 10 days post senescence induction. Scale bar = 50 uM. The proportion of SA-β-gal positive cells is indicated on the top right corner. Cells were counted manually and the mean ± SD of three independent experiments is shown for fMB and one experiment for i-MB. i-MB: iPSC-derived myoblasts, fMB: fetal myoblasts. **F)** Relative expression of p16 and p21 in fMB normalized to the control is shown. Mock cells were transduced with an empty lentivirus and serve as control for RAS-transduced cells. Shown is the mean ± SD from 3 biological replicates. Statistical analysis were performed by t-test. *$p < 0.05$; **$p < 0.01$.

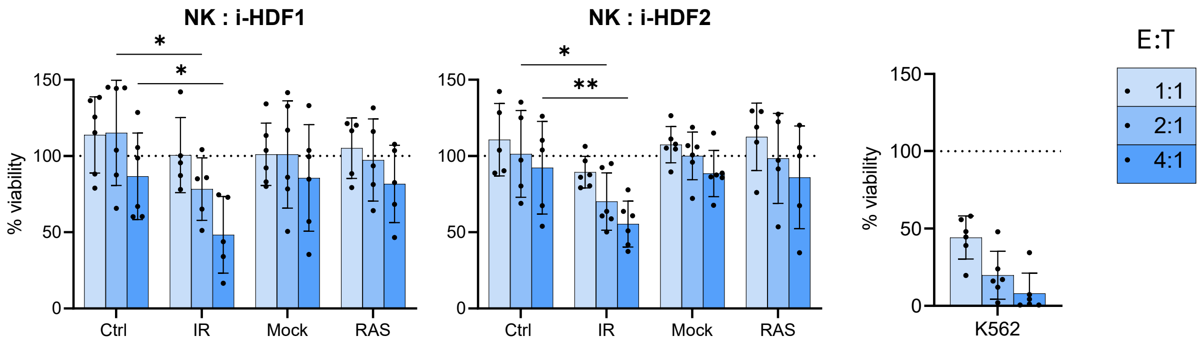

Figure S7. IR-induced senescent iPSC-derived fibroblasts are the target of NK cells. Shown is the viability of i-HDF from two donors after 24 hours of coculture with activated NK cells at varying effector (E) to target (T) cell ratios. Shown is the mean ± SD of n=5-6 independent biological experiments. Cell counts were determined using flow cytometry. Data analysis of viability was performed using two-way ANOVA followed by Šidák's multiple comparisons test. *: $p < 0.05$, **: $p < 0.01$.
