# Supplementary material for: The Immunogenicity of Human Senescent Cells Is Dependent on the Senescence Inducer and Cell Type
Source: Aging Cell. 2026 Feb 12;25(2):e70410. doi: 10.1111/acel.70410 (PMC12900895; doi:10.1111/acel.70410)
Supplement: Supplementary file 2 — Table S1: acel70410‐sup‐0002‐TableS2.docx. [file ACEL-25-e70410-s001.docx]

**Supplementary Table 1:** List of senMAPS evaluated.

| # | **Peptide sequence** | Peptide | | | Protein | | | Protein name |
| --- | --- | --- | --- | --- | --- | --- | --- | --- |
|  |  | NS | IR | RAS | NS | IR | RAS |  |
| #1 | **NFDEKPVII** | 0 | 2 | 1 | 1 | 4 | 2 | ATP-binding cassette sub-family A member 8 OS=Homo sapiens OX=9606 GN=ABCA8 PE=1 SV=3 |
| #2 | **SSWKENVITY** | 0 | 2 | 1 | 24 | 28 | 32 | Sushi von Willebrand factor type A EGF and pentraxin domain-containing protein 1 OS=Homo sapiens OX=9606 GN=SVEP1 PE=1 SV=3 |
| #3 | **AKPVDPIVGY** | 0 | 1 | 1 | 192 | 155 | 248 | Collagen alpha-1(XII) chain OS=Homo sapiens OX=9606 GN=COL12A1 PE=1 SV=2 |
| #4 | **YPKDIYSSF** | 0 | 2 | 15 | 0 | 3 | 69 | Interstitial collagenase OS=Homo sapiens OX=9606 GN=MMP1 PE=1 SV=3 |
| #5 | **YLAPELFVNV** | 0 | 1 | 1 | 0 | 1 | 2 | Receptor-interacting serine/threonine-protein kinase 3 OS=Homo sapiens OX=9606 GN=RIPK3 PE=1 SV=2 |
| #6 | **YLARFLEGL** | 0 | 1 | 1 | 0 | 1 | 1 | Protein Bop OS=Homo sapiens OX=9606 GN=RTL10 PE=1 SV=1 |
| #7 | **ILLDDQFQPKL** | 0 | 1 | 1 | 0 | 1 | 2 | Interleukin-1 receptor-associated kinase 3 OS=Homo sapiens OX=9606 GN=IRAK3 PE=1 SV=2 |
| #8 | **AEQMPQHTL** | 0 | 1 | 1 | 1 | 1 | 1 | Chromodomain-helicase-DNA-binding protein 9 OS=Homo sapiens OX=9606 GN=CHD9 PE=1 SV=2 |
| #9 | **RLLPGDIILKV** | 0 | 2 | 2 | 0 | 2 | 2 | E3 ubiquitin-protein ligase LNX OS=Homo sapiens OX=9606 GN=LNX1 PE=1 SV=1 |
| #10 | **SLFEHFIEL** | 0 | 1 | 5 | 8 | 7 | 6 | WD repeat and FYVE domain-containing protein 3 OS=Homo sapiens OX=9606 GN=WDFY3 PE=1 SV=2 |
| #11 | **ALFEGVVRQI** | 0 | 2 | 6 | 0 | 2 | 14 | GTP-binding protein RAD OS=Homo sapiens OX=9606 GN=RRAD PE=1 SV=2 |
| #12 | **SEDITPRRY** | 0 | 1 | 1 | 8 | 6 | 8 | AMSH-like protease OS=Homo sapiens OX=9606 GN=STAMBPL1 PE=1 SV=2 |
| #13 | **SLAEVLQQL** | 0 | 2 | 3 | 12 | 17 | 22 | All-trans-retinol 13 14-reductase OS=Homo sapiens OX=9606 GN=RETSAT PE=1 SV=2 |
| #14 | **ALLEKLTEL** | 0 | 2 | 2 | 6 | 9 | 9 | DnaJ homolog subfamily C member 13 OS=Homo sapiens OX=9606 GN=DNAJC13 PE=1 SV=5 |
| #15 | **TDKEKAEKLK** | 0 | 4 | 7 | 0 | 37 | 12 | 60S ribosomal protein L38 OS=Homo sapiens OX=9606 GN=RPL38 PE=1 SV=2 |
| #16 | **GLWGQSVPTA** | 1 | 5 | 5 | 1 | 8 | 7 | Tumor necrosis factor receptor superfamily member 10D OS=Homo sapiens OX=9606 GN=TNFRSF10D PE=1 SV=1 |
| #17 | **KVLETLVTV** | 1 | 5 | 2 | 1 | 6 | 2 | HEAT repeat-containing protein 5A OS=Homo sapiens OX=9606 GN=HEATR5A PE=1 SV=2 |
| #18 | **MFNDTLELF** | 0 | 2 | 6 | 3 | 5 | 7 |  |
| #19 | **EEFQKELSQW** | 3 | 5 | 5 | 4 | 7 | 7 | Tuftelin-interacting protein 11 OS=Homo sapiens OX=9606 GN=TFIP11 PE=1 SV=1 |
| #20 | **VLFSSPPVIL** | 0 | 2 | 1 | 5 | 6 | 14 | Major prion protein OS=Homo sapiens OX=9606 GN=PRNP PE=1 SV=1 |
| #21 | **AMAEGKITV** | 4 | 5 | 7 | 5 | 16 | 15 | Periplakin OS=Homo sapiens OX=9606 GN=PPL PE=1 SV=4 |
| #22 | **YMDQWVPVI** | 4 | 7 | 11 | 21 | 31 | 36 | Growth arrest and DNA damage-inducible protein GADD45 alpha OS=Homo sapiens OX=9606 GN=GADD45A PE=1 SV=1 |
| #23 | **NENINLGKKY** | 5 | 7 | 11 | 7 | 10 | 17 | E3 ubiquitin-protein ligase MYLIP OS=Homo sapiens OX=9606 GN=MYLIP PE=1 SV=2 |
| #24 | **AEIEIVKDL** | 0 | 1 | 3 | 8 | 13 | 34 | Prolyl 4-hydroxylase subunit alpha-1 OS=Homo sapiens OX=9606 GN=P4HA1 PE=1 SV=2 |

**Supplementary Table 2:** Complete serum free differentiation medium (cSFDM) for i-LPC culture

| **Reagent** | **Concentration** | **Supplier reference** |
| --- | --- | --- |
| IMDM medium | 75% | Gibco 12440053 |
| Ham’s F12 medium | 25% | Gibco 11765054 |
| B27 supplement | 1x | Gibco 17504044 |
| N2 supplement | 1x | Gibco 17502048 |
| BSA | 0.075% | Wisent |
| Ascorbic acid | 30mM | Sigma Aldrich A92902 |
| 1-Thioglycerol | 40μM | Sigma Aldrich M6145 |
| Primocin | 100μg/mL | Invivogen |
| CHIR99021 | 3μM | StemCell technologies 72054 |
| FGF7 | 10ng/mL | R&D systems 251-KG |
| Dexamethasone | 50nM | Sigma Aldrich D4902 |
| cAMP | 100μM | Sigma Aldrich B7880 |
| IBMX | 100μM | StemCell technologies 72762 |

**Table S3:** List of antibodies

| **Antibody** | **Supplier reference** | **Dilution** | **Application** |
| --- | --- | --- | --- |
| CD31 APC (WM59) | Biolegend 303116 | 1:200 | FACS |
| CD144 PE (BV9) | Biolegend 348506 | 1:200 | FACS |
| Desmin (D33) | Dako M0760 | 1:50 | IF |
| Myosin heavy chain (MHC) (MF20) | Developmental Studies Hybridoma Bank, University of Iowa | 1:100 | IF |
| Surfactant Protein C (SP-C) (H-8) | SCBT (sc-518029) | 2.5μg/mL | IF |
| NKX2-1 (2054B) | R&D systems MAB9458 | 3μg/mL | IF |
| Von Willebrand factor (vWf) | Sigma F3520 | 1:200 | IF |
| CD47 Pacific blue (CC2C6) | Biolegend 323127 | 1:200 | FACS |
| CD26 PE Cy7 (BA5b) | Biolegend 302714 | 1:200 | FACS |
| hCD4 (RPA-T4) | Biolegend 300502 | 1:200 | IF |
| hCD8a (RPA-T8) | Biolegend 301002 | 1:200 | IF |
| hCD3 APC (HIT3a) | Biolegend 300312 | 1:200 | FACS |
| hCD56 BV786 (NCAM16.2) | BD 564058 | 1:200 | FACS |
| hCD69 PE (FN50) | Biolegend 985202 | 1:200 | FACS |
| mCD45 PE cy7 (30-F11) | BD 552848 | 1:200 | FACS |
| hCD45 BUV395 (HI30) | BD 563792 | 1:200 | FACS |
| hCD4 BB515 (RPA-T4) | BD 564419 | 1:200 | FACS |
| hCD8 BV421 (RPA-T8) | BD 562428 | 1:200 | FACS |
| hCD19 PE-CF594 (HIB19) | BD 562294 | 1:200 | FACS |
| PD-L1 APC (29E2A3) | Biolegend 329708 | 1:200 | FACS |
| PD-L2 PE (MIH18) | Biolegend 345506 | 1:200 | FACS |
| HLA-1 BV711 (G46-2.6) | BD 565333 | 1:200 | FACS |
| MICA-B APC (6D4) | Biolegend 320908 | 1:200 | FACS |
| CD112 PE (TX31) | Biolegend 337410 | 1:200 | FACS |
| HLA-E PE Cy7 (3D12) | Biolegend 342608 | 1:200 | FACS |
| CD73 APC (AD2) | Biolegend 344006 | 1:200 | FACS |
| CD155 PE (SKIL4) | Biolegend 337610 | 1:200 | FACS |
| IFN-y unconjugated (NIB42) | BD 551221 | 1:1000 | Elispot |
| CD3 unconjugated (HIT3a) | BD 555336 | 1:50 | Elispot |
| IFN-y unconjugated (R4-6A2) | BD 551216 | 1:1000 | Elispot |
| HLA ABC PE (W6/32) | Biolegend 311405 | 1:200 | FACS |
| Zombie Aqua Dye | Biolegend 77143 | 1:1000 | FACS |
| APC mouse IgG2b κ Isotype Ctrl (MPC11) | Biolegend 400322 | 1:200 | FACS |
| PE mouse IgG1 κ Isotype Ctrl (MOPC-21) | Biolegend 400112 | 1:200 | FACS |
| BV711 mouse IgG1 κ Isotype Ctrl (X40) | BD 563044 | 1:200 | FACS |
| APC mouse IgG2a κ Isotype Ctrl (MOPC-173) | Biolegend 400220 | 1:200 | FACS |
| PE Cy7 mouse IgG1 κ Isotype Ctrl (MOPC-21) | Biolegend 400126 | 1:200 | FACS |
| APC mouse IgG1 κ Isotype Ctrl (MOPC-21) | Biolegend 400122 | 1:200 | FACS |
| PB mouse IgG1 κ Isotype Ctrl (MOPC-21) | Biolegend 400131 | 1:200 | FACS |

**Supplementary Table 4:** List of all senMAPS.
